# Computational Micromechanics Investigation of Percolation and Effective Electro-Mechanical Properties of Carbon Nanotube/Polymer Nanocomposites using Stochastically Generated Realizations: Effects of Orientation and Waviness

**DOI:** 10.3390/polym14235094

**Published:** 2022-11-23

**Authors:** Krishna Kiran Talamadupula, Gary Seidel

**Affiliations:** 1 Western Digital Corporation, 5601 Great Oaks Pkwy, San Jose, CA 95119, USA; 2 Kevin T. Crofton Department of Aerospace and Ocean Engineering, Virginia Polytechnic Institute and State University, Blacksburg, VA 24061, USA

**Keywords:** carbon nanotube, nanocomposite, electrical tunneling, piezoresistivity, gage factor, percolation, realization

## Abstract

The electrical and mechanical properties of carbon nanotube/polymer nanocomposites depend strongly upon several factors such as CNT volume fraction, CNT alignment, CNT dispersion and CNT waviness among others. This work focuses on obtaining estimates and distribution for the effective electrical conductivity, elastic constants and piezoresistive properties as a function of these factors using a stochastic approach with numerous CNT/polymer realizations coupled with parallel computation. Additionally, electrical percolation volume fraction and percolation transitional behavior is also studied. The effective estimates and percolation values were found to be in good agreement with experimental works in the literature. It was found that with increasing CNT volume fraction, the mechanical properties improved. However, due to the interaction of CNTs with one another through electrical tunneling, the conductivity and piezoresistivity properties evolved in a more complex manner. While the degree of alignment played a strong role in the effective properties making them anisotropic, the effect of waviness was found to be insubstantial.

## 1. Introduction, Motivation and Background

Due to the exceptional properties of CNTs, there is significant interest in the research and development of polymer based nanocomposites with CNTs used as reinforcement or filler material. One such example of an exceptional physical property is the axial tensile modulus with reported values in the range of 500-1000 GPa [1]. Similarly, high CNT tensile strengths have also been reported [2]. There have been numerous studies investigating the enhancement of the mechanical properties of CNT reinforced polymer systems [3,4]. Furthermore, several studies have reported on the enhancement of the electrical and electromechanical properties in CNT reinforced polymer systems [5,6]. Liu and Kumar [1] define the phenomenon of electrical percolation as the development of electrically conductive networks resulting from relatively more conductive fillers (for example, CNTs or graphene) within an insulating medium such as a polymer matrix. Bauhofer and Kovacs [7] compiled the values of electrical percolation threshold concentrations reported for various types of CNT and polymers from several different studies. They report finding a wide range of percolation values found in the literature, with values as low as 0.0021 wt.% and as high as 5 wt.% for multi-walled carbon nanotubes (MWCNTs) in epoxy. The mean of the reported percolation values was found to be 0.61 wt.%. A primary motivating factor for the study of electrical percolation is its importance in determining CNT/polymer nanocomposite effective piezoresistivity [8]. There have been several works to this effect using theoretical, computational or experimental analysis methods [5,6,9,10,11,12,13,14,15,16,17,18]. CNT alignment has been found to have a strong influence on both percolation and the piezoresistive response of polymer/CNT nanocomposites [19]. In addition to alignment or a lack thereof, another important characteristic of CNTs in polymer systems is CNT waviness. Wavy, i.e., non-straight or curled, CNTs have been observed in almost all experimental imaging endeavors of CNT/polymer nanocomposites [20,21]. Zare and Rhee [22] concluded that CNT waviness caused only ’moderate’ variations in the electrical conductivity of CNT/polymer nanocomposites as opposed to some other main factors such as filler volume fraction and tunneling distance among others.

The work herein focuses on obtaining effective estimates for and performing statistical analysis of the electrical (conductivity and piezoresistive coefficients) and mechanical (stiffness coefficients) properties of CNT reinforced polymer nanocomposites by controlling the degree of alignment, CNT waviness and CNT volume fraction. The objective is to understand the effects of these factors on the distribution in percolation concentration, electrical conductivity and piezoresistivity. The different degrees of alignment are studied through three different orientation conditions of aligned, imperfectly aligned and randomly oriented. For the imperfectly aligned cases, the CNTs are only allowed to be at angle of $\pm 15^{\circ }$ with respect to the alignment direction. The model developed herein comprises of two key portions—(i) the development of a library of microscale realizations measuring 5μm×5μm in size, where each realization undergoes a randomized CNT seeding process with varying orientation distribution and degree of waviness, and (ii) an electrical and mechanical coupled finite element based computational framework that analyzes the realizations and provides volume averaged properties for the mechanical stiffness coefficients, electrical conductivity coefficients and piezoresistive coefficients. A large number of realizations are generated at each volume fraction. The range of volume fractions considered are between 0.08% till a maximum simulated value of 5.04% corresponding to the range of observed percolation concentrations of weight percents between 0.01 wt.% and 5 wt.%.

The costs involved in generating large sets of specimens experimentally in order to study the percolation behavior of CNT/polymer nanocomposites is prohibitively high. Therefore, this study aims to develop a stochastic representation of the phenomenon of percolation. This includes capturing the transitional response from low volume fraction unpercolated realizations to high volume fraction percolated ones. Scripting together with high performance computers are used to generate the stochastic data distribution developed from a large library of many different realizations. Statistical measures of the effective material properties such as the mean, standard deviation, skewness and kurtosis are presented as a function of the increasing CNT volume fraction. The effective electrical conductivity as a function of volume fraction is assessed in terms of establishing both the onset and completion of percolation transition. The estimates for piezoresistive coefficients are validated by comparison with experimentally reported gage factors. The present study builds upon a closely related initial work by Talamadupula and Seidel [8] that focused on the statistical characterization of these same properties for the special case of aligned, straight CNTs. Results from Talamadupula and Seidel [8] are presented here for comparison purposes with the imperfectly aligned, randomly oriented results and also results with varying degrees of waviness. A few modeling details repeated from [8] and some Supplementary Data with discussion are presented in the Supplementary Materials. The following articles have been referenced in the Supplementary Materials [8,23,24,25,26].

## 2. Modeling Strategy

The mathematical and model details have been presented in Talamadupula and Seidel [8], which analyzed in detail the estimation and statistical distribution of electrical and mechanical properties of CNT/polymer nanocomposites with only aligned, straight CNTs. The current work builds upon the existing modeling framework to extend the analysis to imperfectly aligned, randomly oriented and wavy CNTs. What follows is a brief summary of the key equations and model descriptions applicable herein.

### 2.1. Tunneling Resistance Model

**Figure 1:**
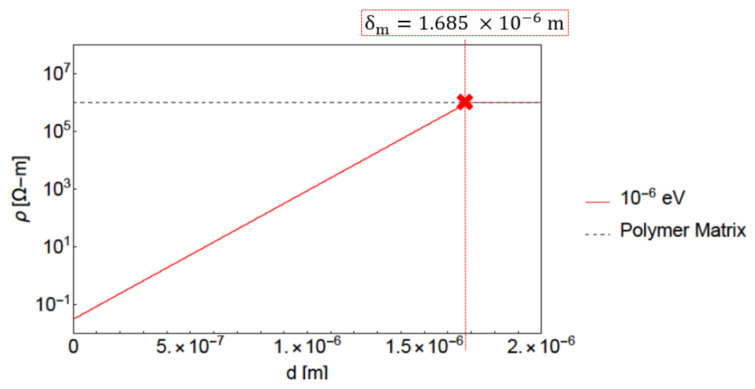
Plot of resistivity ρ vs. tunneling distance *d* on a log-linear scale for barrier potential $\lambda \;=\;10^{-6}\;\mathrm{eV}$.

CNT/polymer nanocomposites have been shown to demonstrate macroscale piezoresistivity stemming from physical mechanisms occurring at different length scales. One of these mechanisms, known as electrical tunneling, is a quantum phenomenon characterized by electrons jumping between conducting surfaces (in the context of CNT/polymer nanocomposites, between CNTs) when separated by an insulating medium (polymer matrix) [27,28]. From Talamadupula and Seidel [8], the tunneling resistivity law is given by

(1) $$\rho_{t}=\frac{h^{2}}{e^{2}\sqrt{2m\lambda }}exp\left(\frac{4\pi d}{h}\sqrt{2m\lambda }\right)$$

where $\rho_{t}$ represents the tunneling resistivity, *e* is electric charge, *d* is the shortest distance between the CNTs, *h* is Planck’s constant, *m* is equal to the mass of an electron and λ signifies the barrier potential of the polymer. λ is a crucial parameter that dictates the strength of the electrical tunneling effect. The relationship between the tunneling resistivity $\rho_{t}$ and the tunneling distance *d* is illustrated in Figure 1.

**Figure 2:**
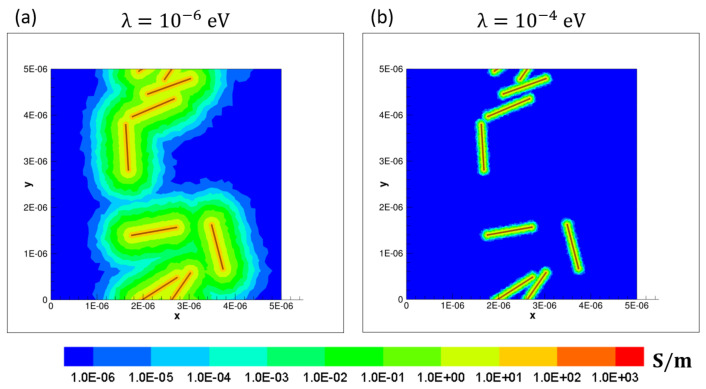
Contour plots of local conductivity for a specific 5μm×5μm realization with randomly oriented CNTs at a volume fraction of 0.56% using (**a**) barrier potential $\lambda =10^{-6}\;\mathrm{eV}$ (percolated case) and (**b**) barrier potential $\lambda =10^{-4}\;\mathrm{eV}$ (unpercolated case).

At every location within the polymer of the CNT/polymer nanocomposite system, the local electrical conductivity is enhanced (i.e., resistivity diminished) according to the tunneling resistivity model. The tunneling distance *d* is computed as twice the minimum distance to any CNT [8]. With increasing *d*, the value of resistivity obtained through the tunneling resistivity equation becomes larger. However, at the maximum tunneling distance $\delta_{m}$, the resistivity obtained from the tunneling resistivity becomes higher than that of the pristine polymer. Beyond this value of tunneling distance the original unmodified value of polymer resistivity is retained, as noted by the plateau region in Figure 1. The model described herein results in the presence of ’fuzzy regions’ [8] which represents a polymer region that surrounds a CNT where the conductivity is increased as per the tunneling model. The maximum tunneling distance $\delta_{m}$ can be visualized as the boundary of these fuzzy regions, where the tunneling distance *d* equals $\delta_{m}$. Figure 2 demonstrates such fuzzy regions for a particular realization having randomly oriented straight CNTs. The effect of the barrier potential λ can also be visualized in Figure 2, with the lower value of λ leading to larger fuzzy regions due to larger $\delta_{m}$. This leads to Figure 2a being percolated in the *y* direction but Figure 2b is not percolated even though the arrangement of CNTs is the same in both cases. It should be noted that the enhancement in conductivity decreases exponentially as the tunneling distance *d* increases.

### 2.2. Material Properties

The material properties are the same as those used in [8]. The electrical and mechanical properties of the CNT and the polymer matrix are assumed as isotropic. It should be noted that the electrical conductivity of the polymer region surrounding the CNTs varies according to the tunneling model. All CNTs are considered to be uniform in length and diameter, with a CNT length of 1μm and CNT diameter of 20nm [29]. The CNTs are assumed to be perfectly straight and aligned for the aligned, imperfectly aligned and randomly oriented cases with straight CNTs. However, the case with waviness treats these CNTs as wavy fibers whose waviness is characterized with a waviness function. A perfect interface is assumed between the CNTs and polymer, i.e., continuity of tractions and displacements across the interface is enforced. It should be noted that the electrical and mechanical properties of CNT/polymer nanocomposites are influenced by the imperfect interface found between the CNT and the polymer [30]. However, some studies report that the assumption of a perfect interface is reasonable [31], with this assumption being widely adopted in many computational and theoretical works that study the effective properties of CNT reinforced nanocomposites [32].

CNT electrical conductivity is taken as $10^{3}\;S/m$ and the conductivity of the epoxy is taken as $10^{-6}\;S/m$. The initial study [8] found that a barrier potential $\lambda =10^{-6}\;\mathrm{eV}$ yielded results which best match other experimental and modeling characterization efforts in terms of electrical percolation, conductivity enhancement and effective nanocomposite piezoresistivity for aligned CNT/polymer nanocomposites. As such, the present study uses this value of $\lambda =10^{-6}\;\mathrm{eV}$ for the tunneling resistivity model for the majority of the results. However, later in the study, the effect of different values of barrier potential for randomly oriented straight CNTs is investigated and compared to experimental studies in the literature. In the present study, the CNT inherent piezoresistive coefficient is selected in order to correspond to a piezoresistive gage factor of 2900 [33]. The value of 2900 is converted to an inherent CNT piezoresistive coefficient using a general form for the gage factor for a uniaxial test [26,34]. The relation has been shown in [8] to be

(2) $$\Pi =\frac{\frac{\Delta \rho }{\rho^{0}}}{\varepsilon_{0}}=\frac{1}{\varepsilon_{0}}\left(\frac{\left(G\varepsilon_{0}+1\right){\left(1-\nu \varepsilon_{0}\right)}^{2}}{1-\varepsilon_{0}}-1\right)$$

where ν is the Poisson’s ratio, $\varepsilon_{0}$ is the applied axial strain, Δρ refers to the change in the resistivity, $\rho^{0}$ is the initial resistivity and G represents the gage factor. For the 2D representation of CNTs in the present work, the piezoresistivity has been taken to be isotropic such that the local resistivity varies in accordance with

(3) $$\rho =\rho_{0}\left(1+\Pi \varepsilon^{a}\right)$$

where Π here represents the coefficient of nanoscale effective piezoresistivity of either the CNT or the polymer matrix, $\varepsilon^{a}$ is the averaged strain, $\rho_{0}$ represents the unstrained resistivity at initial condition and ρ is the resistivity at a point at the current time. It should be noted that since epoxy does not have an inherent piezoresistive effect, its conductivity remains the same when mechanical loading is applied, i.e., Π=0. These properties are summarized in Table 1.

### 2.3. Library of Realizations

**Figure 3:**
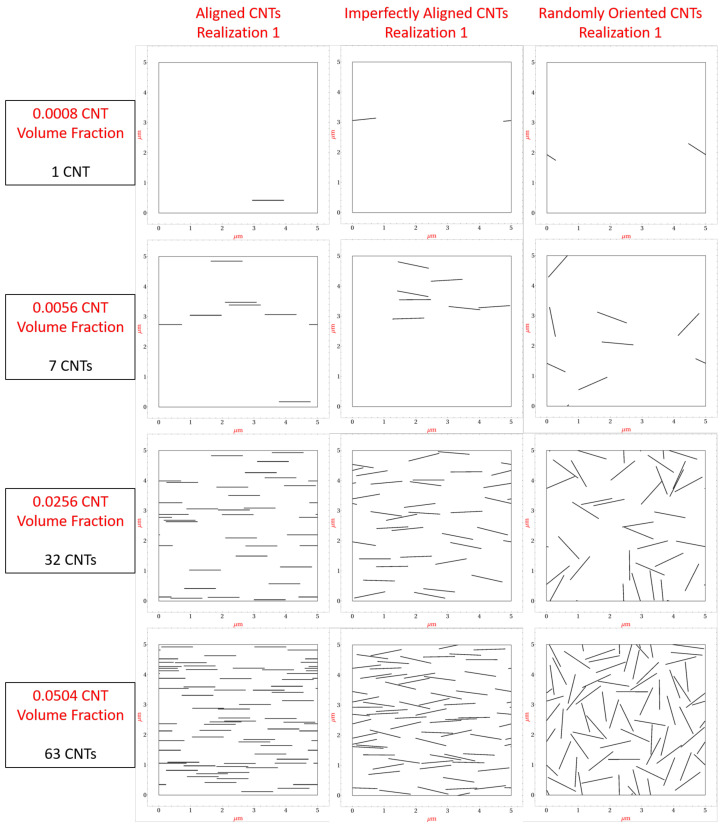
First realization generated for CNT volume fractions 0.08%, 0.56%, 2.56% and 5.04% for the orientation conditions of aligned, imperfectly aligned and randomly oriented CNTs.

A library of numerous 5μm×5μm realizations with random seeding of CNTs are generated with CNT volume fractions of 0.08%, 0.16%, 0.24%, 0.32%, 0.40%, 0.56%, 1.04%, 1.52%, 2.00%, 2.56%, 3.04%, 3.52%, 4.00%, 4.56% and 5.04%. These volume fraction values correspond to 1, 2, 3, 4, 5, 6, 7, 13, 19, 25, 32, 38, 44, 50, 57 and 63, CNTs, respectively. Figure 3 shows the first realization generated for 0.08%, 0.56%, 2.56% and 5.04% CNT volume fractions, respectively, for the aligned, imperfectly aligned and randomly oriented case (with straight CNTs), respectively. The imperfectly aligned case has CNTs generated such that the angle made by the long axis of the CNT with the alignment direction (${\tilde{x}}_{1}$) is at maximum $\pm 15^{\circ }$. The present work models misalignment of $\pm 15^{\circ }$ to capture the partial alignment found in fabricated real world samples [35].

For the randomly oriented case, a random angle is selected for each CNT. At each CNT volume fraction 130 different realizations are generated. The process of randomized CNT seeding and generation of realizations for aligned CNTs is described in [8]. The procedure generalizes to all the cases developed in the present study with the additional steps of including a randomized CNT orientation angle for each seeded CNT (for imperfectly aligned and randomly oriented cases), and incorporation of CNT waviness (for the wavy CNT cases). The CNT seeding process ensures periodicity by allowing for CNTs to reflect across the sides of the domain. Additionally, even the fuzzy regions are reflected in a similar manner. This ensures the periodicity of the electrical tunneling effect. The generated realizations are meshed using 3-noded finite elements. The average number of degrees of freedom increased from around 30,000 to around 100,000 as the CNT volume fraction increased from 0.08% to a maximum of 5.04%. An in-house electro-mechanical 2D finite element code is used to compute the effective electrical and mechanical properties for each realization. This code was previously developed in the Fortran and has been used successfully in several previous works studying CNT/polymer nanocomposite piezoresistivity [8,25,26,34,36,37,38,39] and strain/damage sensing of CNT embedded polymer based energetic materials [18,40,41,42,43].

It should be noted that similar to the procedure followed in [8], there are no specific instructions in the code controlling the CNT seeding process to account for particular levels of agglomeration or dispersion in any region. In other words, there is no biasing in the randomized seeding algorithm. Local regions of clustered/agglomerated CNTs or polymer rich regions are a purely stochastic result. Therefore, from an entirely stochastic process, some dispersions can come to be viewed as agglomerated while others may be viewed as well dispersed depending upon the seeding process.

Furthermore, similar to the previous work [8], the present work regards the generated realizations as ensemble unit cells. This means that these realizations are meant to represent different 5μm×5μm sub domains of the nanocomposite microstructure. The domains are not to be considered as representative sub units of the nanocomposite with CNTs being well dispersed. The effective estimates derived in the present work are ensemble averages (mean μ) of each property across all the generated realizations at each CNT volume fraction. Other statistical measures are also reported such as standard deviation σ, coefficient of variation (standard deviation/mean or σ/μ), skewness and kurtosis of the ensemble data. These statistical measures are used in determining percolation.

It is to be noted that the 2D dimensionality of the problem limits the number of conductive pathways that can be obtained compared to 3D dimensionality, i.e., for a single dimension, if the number of states that the CNTs can be arranged in a unit domain is *N*, then the number of states grows on the order of $N^{2}$ in 2D and $N^{3}$ in 3D. Thus, with the same barrier potential λ, the predicted electrical properties in 2D will be an underestimation compared to the same prediction in 3D. However, the mechanical properties are not expected to change significantly between 2D and 3D models since the CNTs do not interact with one another through electrical tunneling in the same way.

### 2.4. Incorporating CNT Waviness

There have been several works that have modeled the waviness of CNTs explicitly while studying CNT based composite systems. Some have modeled the shape of the CNTs using sinusoidal or bow shaped idealizations [20,21,44,45,46]. In the present study, CNT waviness is achieved with Lagrange shape functions assuming a 4-noded 1D element which is mapped onto the global domain as the actual shape of the CNT. This framework allows for more variation in the nature of CNT waviness than a relatively simpler sinusoidal shape mapping which can at most have two parameters, i.e., frequency and amplitude unless further parameterized.

The methodology used here is similar to many finite element textbooks which employ Lagrange shape functions to develop basis functions for 1D finite elements. It should be noted that the shape functions discussed here are only used to randomly generate a waviness function and have no relation to the implementation of the finite element solution scheme to obtain effective properties. Considering a master element $\Omega_{m}$ with n nodes with their corresponding local coordinates $s_{1},s_{2},\ldots ,s_{n}$. The domain of the master element $\Omega_{m}$ is limited to [−1, 1] such that $s_{1}=-1$ and $s_{n}=1$. It is required that the shape functions $N_{i}\left(s\right)$ vanish at every other node apart from *i*, suggesting that the shape functions $N_{i}\left(s\right)$ take the form

(4) $$N_{i}\left(s\right)=c\left(s-s_{1}\right)\left(s-s_{2}\right)\ldots \left(s-s_{i-1}\right)\left(s-s_{i+1}\right)\ldots \left(s-s_{n}\right)$$

where *c* is a constant. This expression can be evaluated by requiring $N_{i}\left(s\right)=1$ for $s=s_{i}$ and thus

(5) $$N_{i}\left(s\right)=\frac{\left(s-s_{1}\right)\left(s-s_{2}\right)\ldots \left(s-s_{i-1}\right)\left(s-s_{i+1}\right)\ldots \left(s-s_{n}\right)}{\left(s_{i}-s_{1}\right)\left(s_{i}-s_{2}\right)\ldots \left(s_{i}-s_{i-1}\right)\left(s_{i}-s_{i+1}\right)\ldots \left(s_{i}-s_{n}\right)}$$

For a 4-noded element the Lagrange shape function is evaluated as

(6) $$\begin{array}{c} N_{1}\left(s\right)=-\frac{1}{16}\left(9s^{3}-9s^{2}-s+1\right) \end{array}$$

(7) $$\begin{array}{c} N_{2}\left(s\right)=+\frac{9}{16}\left(3s^{3}-s^{2}-3s+1\right) \end{array}$$

(8) $$\begin{array}{c} N_{3}\left(s\right)=-\frac{9}{16}\left(3s^{3}+s^{2}-3s-1\right) \end{array}$$

(9) $$\begin{array}{c} N_{4}\left(s\right)=+\frac{1}{16}\left(9s^{3}+9s^{2}-s-1\right) \end{array}$$

**Figure 4:**
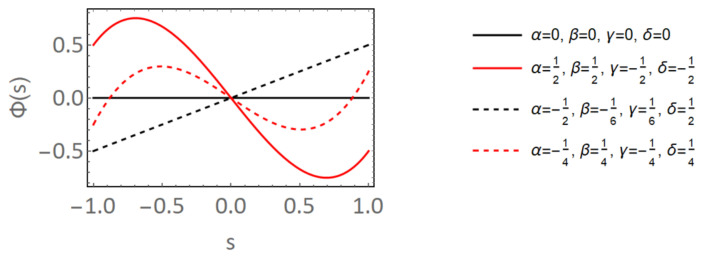
Wavy CNT shapes generated by selection of coefficients of waviness generation function Φ(s).

The shape of a CNT in the master element space is dictated by

(10) $$\Phi \left(s\right)=\alpha N_{1}\left(s\right)+\beta N_{2}\left(s\right)+\gamma N_{3}\left(s\right)+\delta N_{4}\left(s\right)$$

where α, β, γ and δ are randomly chosen coefficients between [−1, 1]. A few examples of different CNT shapes based on the selection of these coefficients are presented in Figure 4, from which it can be observed that the degree of CNT waviness can vary drastically between no waviness (straight CNTs—black lines) and significant waviness (red lines) depending upon the selection of coefficients α, β, γ and δ. A 4-noded shape function was selected so as to allow for at least two inflection points since CNTs are known to exhibit multiple changes in the sign of its curvature along its length. The waviness of CNTs is measured with tortuosity, the property of a curve to be tortuous or winding. In its simplest form, tortuosity τ is defined as the ratio of the length of the curve *C* to the distance between its ends *L* such that τ=C/L, where a straight line yields τ=1 and a completely closed curve yields τ→∞. τ values for the four curves shown in Figure 4 are 1, 1.315, 1 and 1.337 in order, respectively.

**Figure 5:**
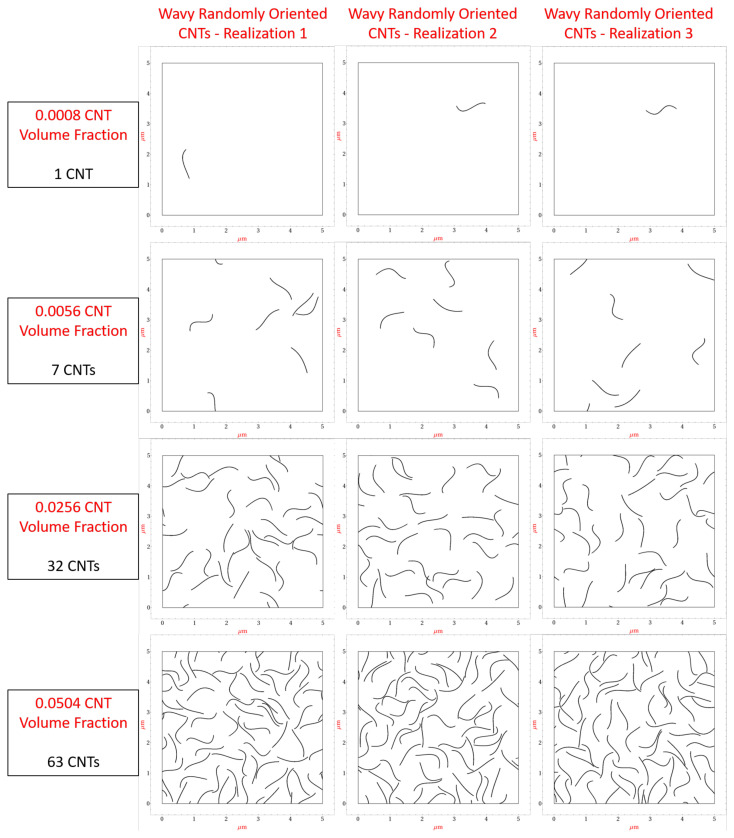
First three realizations generated for CNT volume fractions 0.08%, 0.56%, 2.56% and 5.04% for the randomly oriented orientation condition with wavy CNTs.

In the present study, the maximum amount of tortuosity allowed for any CNT shape is restricted to 1.2 so as to simulate small amounts of CNT waviness. After completely generating Φ(s) and making sure τ≤1.2, the CNT shape is mapped into the global domain (5μm×5μm realization) at the randomly selected seed point taking into account rotation of the CNT based on the selected orientation angle for that CNT. During the waviness generation process, if τ>1.2, then a new waviness function Φ(s) is randomly generated with a new set of randomly selected coefficients α, β, γ and δ with the process repeating until τ≤1.2 is obtained. Since the length of the curve *C* varies depending upon the selection of coefficients α, β, γ and δ, the waviness function Φ(s) is scaled appropriately when mapping the CNT shape into the global domain so as to maintain the CNT length at 1μm, ensuring that the volume of a straight CNT and a wavy CNT (with any degree of tortuosity) is the same. A library of realizations is generated for randomly oriented wavy CNTs similar to the aligned, imperfectly aligned and randomly oriented cases with straight CNTs for the same range of CNT volume fractions. Figure 5 shows the first 3 realizations generated for 0.08%, 0.56%, 2.56% and 5.04% CNT volume fractions, respectively.

### 2.5. Problem Setup and Computing Effective Estimates

The governing equations, application of boundary conditions and method to calculate effective stiffness, conductivity and piezoresistivity components was extensively discussed in the initial sister study [8]. An abridged version is presented in the Supplementary Materials for the benefit of the reader. Specifically with regards to piezoresistivity, it is important to note that macroscale gage factors are generally reported as opposed to mascroscale piezoresistivity. As such, the effective piezoresistive coefficients determined in the present work will be converted into gage factors to allow for comparison with other works. These gage factors $G_{1}^{Eff}$ and $G_{2}^{Eff}$ (in the ${\tilde{x}}_{1}$ and ${\tilde{x}}_{2}$ direction, respectively) can be determined [8] as

(11) $$\begin{array}{c} G_{1}^{Eff}=\Pi_{11}^{Eff}\left(1+{\tilde{\varepsilon }}_{0}\right)+1 \end{array}$$

(12) $$\begin{array}{c} G_{2}^{Eff}=\Pi_{22}^{Eff}\left(1+{\tilde{\varepsilon }}_{0}\right)+1 \end{array}$$

Measures of gage factors corresponding to terms $\Pi_{12}^{Eff}$, $\Pi_{21}^{Eff}$, $\Pi_{66}^{Eff}$, $\Pi_{16}^{Eff}$, $\Pi_{26}^{Eff}$, $\Pi_{61}^{Eff}$ and $\Pi_{62}^{Eff}$ are typically not reported in the literature.

#### Solution Scheme and Algorithm

**Figure 6:**
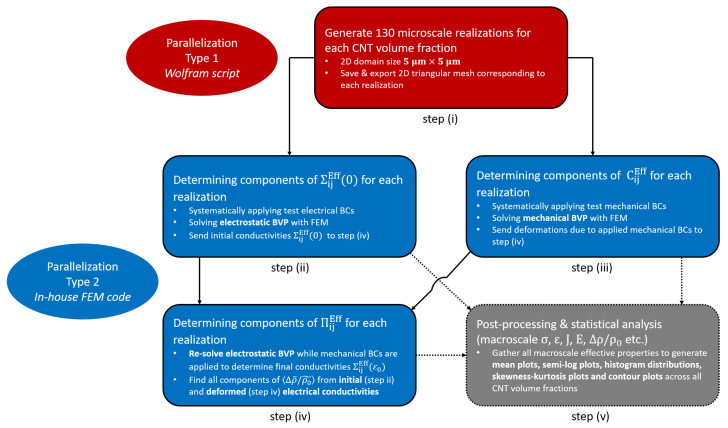
Solution scheme flowchart.

A flowchart algorithm describing the step-wise methodology used in this study is shown in Figure 6. The algorithm used herein is the same as the one used in [8]. In order to reduce the vast amount of computation time required if the computations were done serially, two different levels of parallelization were employed, type 1 and type 2. The first parallelization type is used in step (i) where the wolfram script is used to generate 130 realizations at every volume fraction and for each CNT orientation case. The next parallelization type is used for steps (ii) to (iv) where a previously developed finite element method program. The coupled electrical and mechanical BVP is solved for all realizations under all the test boundary conditions shown in Tables S1 and S2. This results in obtaining the components of the macroscale $\Sigma_{ij}^{Eff}$, $C_{ij}^{Eff}$ and $\Pi_{ij}^{Eff}$ tensors. Both parallelization types are coupled together with the help of high performance computers to improve computation speed. Furthermore, ’GNU parallel’, a shell tool for executing jobs in parallel using one or more computers [47], is used to direct the parallelized tasks, further improving computation speed.

For every orientation case and volume fraction, a total of 224 processors across 14 compute nodes were used in parallelization type 1 for the generation of a 130 realizations. In the aligned study [8], computation time reduced by a factor of approximately 100 due to the implementation of type 1 parallelization for the maximum volume fraction simulated of 5.04%. In the present work, this reduction factor increased to approximately 1000 for the randomly oriented straight case and the randomly oriented wavy case. This is because of the sequential process of seeding CNTs. A new CNT is seeded into the domain only if it does not interpenetrate with any other CNT that has already been seeded. As a result, it becomes harder for CNT seeding to occur when CNTs have randomized alignment as opposed to being perfectly aligned (or even imperfectly aligned), i.e., the algorithm requires more iterations to randomly generate a CNT (with its seed location, orientation angle and waviness morphology) that can fit in the increasingly crowded 2D domain. As such, the benefits of implementing parallelization in terms of decreasing walltime increases with the increase in the randomness of CNT orientation. For the maximum volume fraction simulated of 5.04% for the randomly oriented wavy case, the walltime was approximately 9 h as opposed to an estimated walltime of approximately 300 days through serial execution of code. Parallelization type 2 showed similar improvements in computational efficiency as was observed in [8]. For every orientation case and volume fraction, a total of 48 processors across 3 compute nodes were used to analyze all 130 realizations (The electro-mechanical BVP corresponding to a few of the meshes at high volume fractions cannot be solved due to poor mesh quality, i.e., when 2 CNTs are seeded very close to each other resulting in elements between the two CNTs which are orders of magnitude smaller than the rest of the mesh. However, this occurs for at most 2 realizations per volume fraction per orientation case analyzed in this study so it is unlikely to affect the estimates). For the batch run corresponding to 5.04% CNT volume fraction (the maximum simulated volume fraction), walltime was approximately 3 h. This is a significant improvement over serial execution, which showed an order of magnitude larger walltime of approximately 39 h. The implementation of type 2 parallelization is done independently of type 1 parallelization, i.e., type 2 is executed after type 1 is finished completely. After implementing both types of parallelizations, a post-processing step gathers the effective estimates and generates relevant contour plots, mean plots, semi-log plots, histogram distributions, scatter plots and skewness-kurtosis plots. These are discussed in the results section.

## 3. Results and Discussion

### 3.1. Estimates of Effective Mechanical Stiffness with Straight CNTs

#### 3.1.1. Effect of Orientation

**Figure 7:**
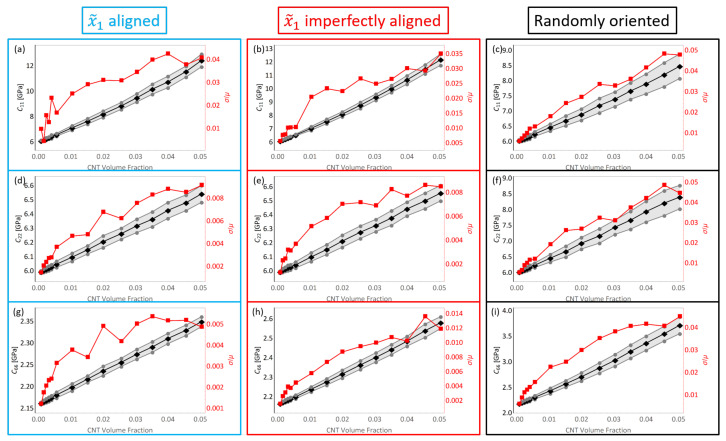
The mean, mean ± standard deviation and coefficient of variation depicted in black, grey and red, respectively, for $C_{11}$ (**a**–**c**), $C_{22}$ (**d**–**f**) and $C_{66}$ (**g**–**i**) for straight ${\tilde{x}}_{1}$ aligned CNTs, straight ${\tilde{x}}_{1}$ imperfectly aligned ($\pm 15^{\circ }$) CNTs and straight randomly oriented CNTs.

The stiffness components estimates for $C_{11}$, $C_{22}$ and $C_{66}$ for all volume fractions for the three different orientation conditions with straight CNTs—${\tilde{x}}_{1}$ aligned, ${\tilde{x}}_{1}$ imperfectly aligned (by $\pm 15^{\circ }$) and randomly oriented CNTs are shown in Figure 7. These plots depict the estimated value (calculated mean) of stiffness as a function of CNT volume fraction. In the present work, these estimate plots depict the mean with black data points and the mean ± standard deviation (μ±σ) with grey data points. The grey shaded in portion that exists between the black lines represents the uncertainty in the estimate. A statistical measure often used to depict deviation is the coefficient of variation, which is the standard deviation divided by the mean (σ/μ). In the estimate plots found in this study, this quantity is plotted with red data points with its axis on the right hand side.

It is observed that the mean (black data points) for all stiffness terms shown in Figure 7 increase with increase in CNT volume fraction regardless of the orientation condition, which is expected since the CNT is several orders of magnitude larger in stiffness than the polymer matrix. The increase in $C_{11}$ is larger than the increase in $C_{22}$ for both the ${\tilde{x}}_{1}$ aligned and ${\tilde{x}}_{1}$ imperfectly aligned cases, with $C_{11}$ increasing from 5.984 GPa corresponding to pure epoxy to 12.415 GPa and 12.168 GPa at 5.04% CNT volume fraction for ${\tilde{x}}_{1}$ aligned and ${\tilde{x}}_{1}$ imperfectly aligned cases, respectively, (an increase of 107.47% and 103.34% for ${\tilde{x}}_{1}$ aligned and ${\tilde{x}}_{1}$ imperfectly aligned cases, respectively). On the other hand, $C_{22}$ increases from 5.984 GPa corresponding to pure epoxy to 6.541 GPa and 6.553 GPa at 5.04% CNT volume fraction for ${\tilde{x}}_{1}$ aligned and ${\tilde{x}}_{1}$ imperfectly aligned cases, respectively, (an increase of 9.31% and 9.51% for ${\tilde{x}}_{1}$ aligned and ${\tilde{x}}_{1}$ imperfectly aligned cases, respectively). The alignment of CNTs in the ${\tilde{x}}_{1}$ direction causes the mismatch in the effective properties in the ${\tilde{x}}_{1}$ and ${\tilde{x}}_{2}$ direction, with the alignment aiding the effective stiffness in the ${\tilde{x}}_{1}$ direction for the ${\tilde{x}}_{1}$ aligned and ${\tilde{x}}_{1}$ imperfectly aligned cases. Furthermore, due to the slight misalignment in the ${\tilde{x}}_{1}$ imperfectly aligned cases, the $C_{11}$ estimates are always slightly lower and the $C_{22}$ estimates are always slightly higher for the ${\tilde{x}}_{1}$ imperfectly aligned cases when compared to the ${\tilde{x}}_{1}$ aligned cases.

The estimates for $C_{11}$ and $C_{22}$ are similar to each other for the randomly oriented cases, increasing from the pure epoxy value of 5.984 GPa to values of 8.473 GPa and 8.394 GPa, respectively, at 5.04% CNT volume fraction (increases of 41.59% and 40.27%, respectively). Since there is no specific alignment of the CNTs in the randomly oriented cases, the effect of mechanical reinforcement provided by the CNTs is equal in the ${\tilde{x}}_{1}$ and ${\tilde{x}}_{2}$ directions and as such, the $C_{11}$ and $C_{22}$ estimates for the randomly oriented cases turn out to be similar. Moreover, these estimates are between the $C_{11}$ and $C_{22}$ estimates obtained for the ${\tilde{x}}_{1}$ aligned and ${\tilde{x}}_{1}$ imperfectly aligned cases.

The estimates for $C_{66}$ are similar for the ${\tilde{x}}_{1}$ aligned and ${\tilde{x}}_{1}$ imperfectly aligned cases, with $C_{66}$ increasing from the pure epoxy value of 2.158 GPa to 2.349 GPa and 2.579 GPa at 5.04% CNT volume fraction for ${\tilde{x}}_{1}$ aligned and ${\tilde{x}}_{1}$ imperfectly aligned cases, respectively, (an increase of 8.85% and 19.51% for ${\tilde{x}}_{1}$ aligned and ${\tilde{x}}_{1}$ imperfectly aligned cases, respectively). The slight degree of misalignment for the ${\tilde{x}}_{1}$ imperfectly aligned case aids in the reinforcement of the shear component. Reinforcement of the shear component through misalignment is illustrated further through the $C_{66}$ estimates for the randomly oriented cases, with $C_{66}$ increasing from the pure epoxy value of 2.158 GPa to 3.713 GPa at 5.04% CNT volume fraction (an increase of 72.06%).

It is noteworthy that for all stiffness terms shown in Figure 7, the coefficient of variation σ/μ increases with increase in CNT volume fraction. However, the maximum value observed for $C_{11}$ at 5.04% CNT volume fraction is relatively small at 0.048 for the case with straight randomly oriented CNTs. Histogram distributions of the mechanical components $C_{11}$, $C_{22}$, $C_{12}$ and $C_{66}$ reveals normally distributed data, which is evident from the small values of the coefficient of variation σ/μ, i.e., σ<<μ. Examples of these plots are shown in the Supplementary Materials at 0.56% CNT volume fraction for the randomly oriented case. For the ${\tilde{x}}_{1}$ aligned case, the deviation in the data results from CNT dispersion and has been reported previously in the initial study [8]. The orientation of CNTs also plays a significant role in generating deviation in the data set in addition to the effect of CNT dispersion. This is evident from the relatively larger coefficient of variation values for the randomly oriented cases for stiffness components $C_{11}$ and $C_{22}$ from Figure 7 when compared to the ${\tilde{x}}_{1}$ aligned and ${\tilde{x}}_{1}$ imperfectly aligned cases.

**Figure 8:**
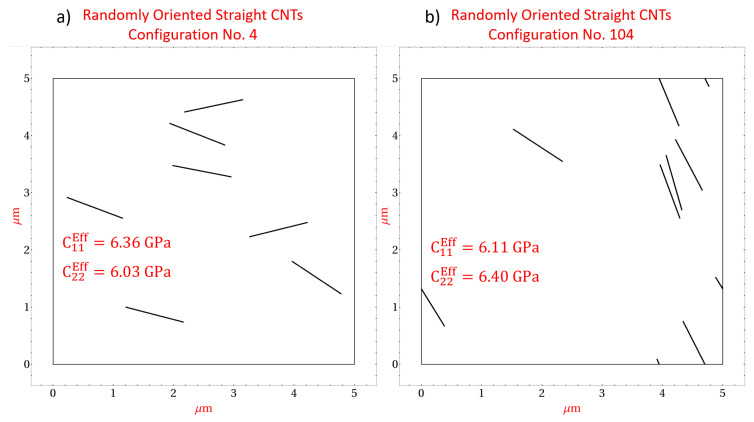
Microscale realizations with straight randomly oriented CNTs at 0.56% CNT volume fraction depicting the effect of CNT orientation on stiffness components $C_{11}$ and $C_{22}$.

**Figure 9:**
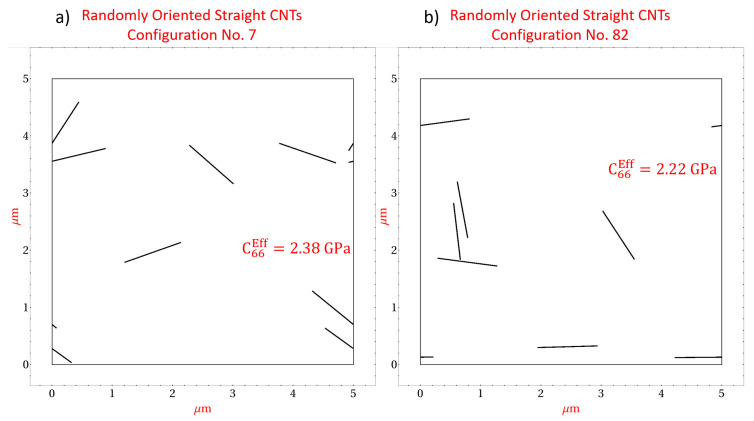
Microscale realizations with straight randomly oriented CNTs at 0.56% CNT volume fraction depicting the effect of CNT orientation on stiffness component $C_{66}$.

Figure 8 presents two specific microscale realizations with straight randomly oriented CNTs at a CNT volume fraction of 0.56%. Figure 8a depicts realization number 4 which yields effective stiffness components $C_{11}^{Eff}=6.36\;GPa$ and $C_{22}^{Eff}=6.03\;GPa$. The discrepancy between $C_{11}^{Eff}$ and $C_{22}^{Eff}$ can be correlated with the orientation of the CNTs within realization number 4, where it is observed that most of the CNTs are oriented more towards the ${\tilde{x}}_{1}$ direction as opposed to the ${\tilde{x}}_{2}$ direction. As such, the mechanical reinforcement provided by the CNTs in response to an applied strain will be greater in the ${\tilde{x}}_{1}$ direction than the ${\tilde{x}}_{2}$ direction resulting in a larger $C_{11}^{Eff}$ compared to $C_{22}^{Eff}$. Figure 8b depicts realization number 104 which yields effective stiffness components $C_{11}^{Eff}=6.11\;GPa$ and $C_{22}^{Eff}=6.40\;GPa$. The discrepancy for this realization arises due to the same reason as realization number 4 but in this case the CNTs reinforce the ${\tilde{x}}_{2}$ direction to a larger extent than the ${\tilde{x}}_{1}$ direction. The effect of CNT orientation on the effective stiffness components can also be visualized for the shear component $C_{66}$ in Figure 9, which presents two microscale realizations, realization number 7 and realization number 82 at 0.56% CNT volume fraction with straight randomly oriented CNTs. Figure 9a yields $C_{66}^{Eff}=2.38\;GPa$ and Figure 9b yields $C_{66}^{Eff}=2.22\;GPa$. The difference in these effective values arises because the CNTs in Figure 9a are oriented such that they are not favorably aligned in either the ${\tilde{x}}_{1}$ or ${\tilde{x}}_{2}$ directions, i.e., CNTs are oriented close to π/4 or 3π/4 radians, where as the CNTs in Figure 9b are oriented such that they are more in alignment with the ${\tilde{x}}_{1}$ or ${\tilde{x}}_{2}$ directions.

**Figure 10:**
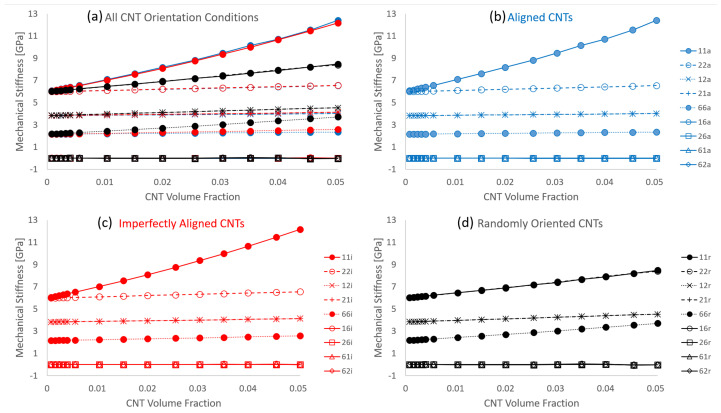
Mean of every stiffness tensor component $C_{ij}$ for (**a**) all 3 different orientation cases with straight CNTs, (**b**) straight ${\tilde{x}}_{1}$ aligned CNTs, (**c**) straight ${\tilde{x}}_{1}$ imperfectly aligned CNTs and (**d**) straight randomly oriented CNTs.

The estimates for stiffness components $C_{16}$, $C_{26}$, $C_{61}$ and $C_{62}$ were reported as being near zero values for the straight aligned CNT case in the initial study [8]. Similarly, these components are also found to be near zero values for the imperfectly aligned and randomly oriented cases where (i) the individual effective values $C_{16}^{Eff}$, $C_{26}^{Eff}$, $C_{61}^{Eff}$ and $C_{62}^{Eff}$ are relatively very small when compared to other terms such as $C_{11}^{Eff}$ and ii) the estimates for $C_{16}$, $C_{26}$, $C_{61}$ and $C_{62}$ when averaged result in a negligibly small value for all CNT volume fractions. Therefore, estimates for these components are concluded to be zero for all CNT volume fractions for all orientation conditions. The estimate of every term of the stiffness tensor $C_{ij}$ for all orientation conditions is presented in Figure 10a with separate plots showing the estimates for just the ${\tilde{x}}_{1}$ aligned, ${\tilde{x}}_{1}$ imperfectly aligned and randomly oriented cases presented in Figure 10b–d, respectively. Figure 10 reinforces key observations made earlier which are i) with increase in the degree of misalignment (first with ${\tilde{x}}_{1}$ imperfectly aligned followed by randomly oriented) at any given CNT volume fraction, the estimate for $C_{11}$ decreases and the estimates for $C_{22}$ and $C_{66}$ increase (ii) at all CNT volume fractions, the estimates for $C_{11}$ and $C_{22}$ for the randomly oriented cases are similar and lie between the $C_{11}$ and $C_{22}$ estimates for the ${\tilde{x}}_{1}$ aligned and ${\tilde{x}}_{1}$ imperfectly aligned cases and (iii) estimates for stiffness terms $C_{16}$, $C_{26}$, $C_{61}$ and $C_{62}$ are negligibly small values at all CNT volume fractions and for all three orientation cases. Furthermore, from Figure 10 it is seen that the stiffness terms $C_{12}$ and $C_{21}$ are nearly identical to each other with these estimates increasing with increase in CNT misalignment at any given CNT volume fraction, which is consistent with the notion of a symmetric stiffness matrix.

#### 3.1.2. Estimates for Engineering Constants

The estimates of the stiffness components for the straight ${\tilde{x}}_{1}$ aligned cases were converted to engineering constants in the initial study [8] assuming that the straight ${\tilde{x}}_{1}$ aligned CNTs/polymer nanocomposite system exhibited transverse isotropy. The 2–3 plane was taken as the plane of isotropy, where ${\tilde{x}}_{1}$ was the alignment direction, ${\tilde{x}}_{2}$ was the transverse direction and ${\tilde{x}}_{3}$ was the out of plane direction. This analysis is extended to the ${\tilde{x}}_{1}$ imperfectly aligned and randomly oriented cases in order to compare the mechanical reinforcement provided by the CNTs between orientation cases and to compare with other reported values in the literature. As such, the obtained estimates for the mechanical stiffnesses $C_{ij}$ are used to determine the standard engineering constants of Young’s modulus *E*, Poisson’s ratio ν and shear modulus *G* for all three orientation conditions. The ${\tilde{x}}_{1}$ imperfectly aligned case is assumed to exhibit transverse isotropy similar to the ${\tilde{x}}_{1}$ aligned cases. This assumption is compatible with the obtained stiffness estimates for the ${\tilde{x}}_{1}$ imperfectly aligned cases where the properties in the ${\tilde{x}}_{1}$ and ${\tilde{x}}_{2}$ direction differ significantly similar to the ${\tilde{x}}_{1}$ aligned case. However, the randomly oriented cases exhibit almost identical properties in the ${\tilde{x}}_{1}$ and ${\tilde{x}}_{2}$ direction in terms of stiffness estimates and hence assuming an isotropic response from the nanocomposite system with randomly oriented CNTs is more appropriate. However, transverse isotropy is assumed even for the randomly oriented cases to check if the obtained engineering constants exhibit isotropy (similar response in ${\tilde{x}}_{1}$ and ${\tilde{x}}_{2}$ direction). A full development of the stiffness matrix and procedure to obtain engineering constants was done in Talamadupula and Seidel [8]. Here, a summarized version is presented. $\nu_{21}$ is related to $\nu_{12}$ through

(13) $$\frac{\nu_{21}}{E_{2}}=\frac{\nu_{12}}{E_{1}}$$

The stiffness matrix for a transversely isotropic material for 2D plane strain conditions is given as

(14) $$\begin{array}{c} \left\{\begin{array}{c} \sigma_{1}\\ \sigma_{2}\\ \sigma_{6} \end{array}\right\}=\left[\begin{array}{ccc} \frac{E_{1}^{2}\left(-1+\nu_{23}\right)}{2E_{2}\nu_{12}^{2}+E_{1}\left(-1+\nu_{23}\right)} & \frac{E_{1}E_{2}\nu_{12}}{E_{1}-2E_{2}\nu_{12}^{2}-E_{1}\nu_{23}} & 0\\ \frac{E_{1}E_{2}\nu_{12}}{E_{1}-2E_{2}\nu_{12}^{2}-E_{1}\nu_{23}} & \frac{E_{2}\left(-E_{1}+E_{2}\nu_{12}^{2}\right)}{\left(2E_{2}\nu_{12}^{2}+E_{1}\left(-1+\nu_{23}\right)\right)\left(1+\nu_{23}\right)} & 0\\ 0 & 0 & 2G_{12} \end{array}\right]\left\{\begin{array}{c} \varepsilon_{1}\\ \varepsilon_{2}\\ \varepsilon_{6} \end{array}\right\} \end{array}$$

**Figure 11:**
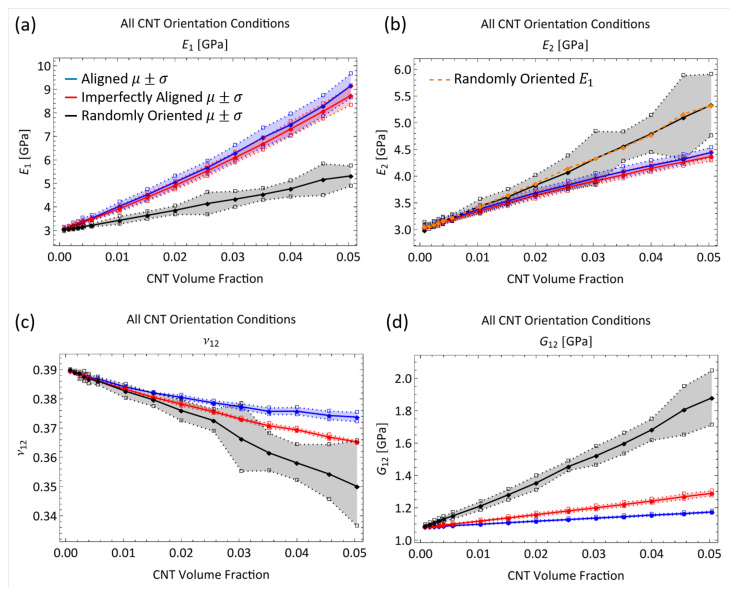
Estimate of engineering constants (**a**) $E_{1}$, (**b**) $E_{2}$, (**c**) $\nu_{12}$ and (**d**) $G_{12}$ for all three orientation conditions with straight CNTs.

The computed engineering constants are presented in Figure 11. Along with the mean value, an upper and lower bound corresponding to μ+σ and μ−σ, respectively, are presented. Both the ${\tilde{x}}_{1}$ aligned and ${\tilde{x}}_{1}$ imperfectly aligned cases show larger mechanical reinforcement in the alignment direction. The Young’s modulus increases by 205.09% and 189.75% from the pure epoxy value of 3 GPa to 9.153 GPa and 8.693 GPa at 5.04% CNT volume fraction for the ${\tilde{x}}_{1}$ aligned and ${\tilde{x}}_{1}$ imperfectly aligned cases, respectively. However, in the transverse direction (${\tilde{x}}_{2}$), the Young’s modulus $E_{2}$ only increases by 48.16% and 48.93% from the pure epoxy value of 3 GPa to 4.445 GPa and 4.468 GPa at 5.04% CNT volume fraction for the ${\tilde{x}}_{1}$ aligned and ${\tilde{x}}_{1}$ imperfectly aligned cases, respectively. Similar to the trends observed for $C_{11}$ and $C_{22}$ between the ${\tilde{x}}_{1}$ imperfectly aligned and ${\tilde{x}}_{1}$ aligned cases at a given CNT volume fraction, the misalignment in the ${\tilde{x}}_{1}$ imperfectly aligned case causes $E_{1}$ to be smaller and $E_{2}$ to be larger than the corresponding values for the ${\tilde{x}}_{1}$ aligned case at each CNT volume fraction. The effect of misalignment can be observed to a much larger degree for the randomly oriented case where both $E_{1}$ and $E_{2}$ are almost identical to one another (observed from Figure 11b), with these values between the $E_{1}$ and $E_{2}$ values for the ${\tilde{x}}_{1}$ aligned and ${\tilde{x}}_{1}$ imperfectly aligned cases. $E_{1}$ and $E_{2}$ for the randomly oriented case increases from 3 GPa (pristine polymer) till 5.297 GPa and 5.239 GPa, respectively, at 5.04% CNT volume fraction (increases of 76.57% and 74.63%, respectively). Since $G_{12}$ and $C_{66}$ are related by $C_{66}=2G_{12}$, the trends in $G_{12}$ are exactly the same as that for $C_{66}$, with the values being higher with increase in the degree of misalignment at a given CNT volume fraction. $G_{12}$ values at 5.04% CNT volume fraction are 1.174 GPa, 1.290 GPa and 1.857 GPa for ${\tilde{x}}_{1}$ aligned, ${\tilde{x}}_{1}$ imperfectly aligned and randomly oriented cases, respectively, (increases of 8.85%, 19.56% and 72.10% from the pristine polymer value of 1.079 GPa). Poisson’s ratio $\nu_{12}$ exhibits a decreasing trend for all three orientation conditions with increasing CNT volume fraction, with the rate of decrease being larger with increased degree of misalignment. $\nu_{12}$ decreases to 0.374, 0.360 and 0.351 at 5.04% CNT volume fraction for the ${\tilde{x}}_{1}$ aligned, ${\tilde{x}}_{1}$ imperfectly aligned and randomly oriented cases, respectively, (decreases of 4.10%, 7.69% and 10.00%, respectively, from the pristine polymer value of 0.39).

#### 3.1.3. Comparison to Other Studies in the Literature

The improvement in the mechanical properties of CNT/polymer nanocomposites has been demonstrated through multiple experimental and modeling studies. The trends observed for the engineering constants for the aligned cases was compared with other characterization efforts in the initial study [8]. In the present work, the engineering constants for the randomly oriented cases are compared with other works in the literature. Since the densities of CNTs ($1.52\;g/cm^{3}$ [1]) and epoxy ($1.4\;g/cm^{3}$) are very similar to each other, the values for volume fractions and weight fractions for CNT/epoxy composites can be expected to be very similar. The relation between the weight fractions and densities can be computed as follows

(15) $$w_{c}=\frac{\rho_{c}V_{c}g}{\rho_{c}V_{c}g+\rho_{m}V_{m}g}$$

where $w_{c}$ is the weight fraction of CNTs, $\rho_{c}$ is the density of CNTs, $V_{c}$ is the volume of CNTs, $\rho_{m}$ is the density of epoxy, $V_{m}$ is the volume of epoxy and *g* is acceleration due to gravity. Simplifying this relation results in

(16) $$w_{c}=\frac{\phi_{c}}{0.92+0.08\phi_{c}}$$

where $\phi_{c}$ is the volume fraction of CNTs.

**Figure 12:**
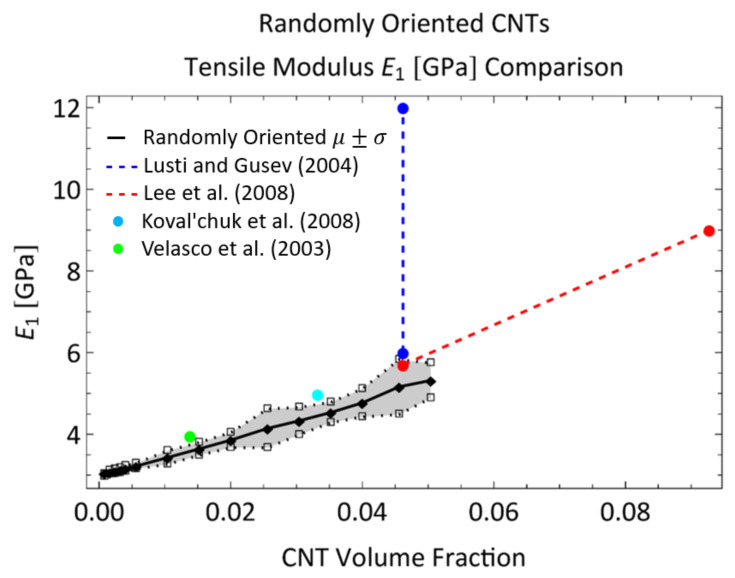
Comparing engineering constant $E_{1}$ for randomly oriented straight CNTs with characterization efforts in the literature [3,49,50,51].

A summary of the improvement in the tensile modulus of CNT/polymer nanocomposites with random dispersions and no specific alignment of CNTs can be found in Rahmat and and Hubert [48] with modeling predictions and experimentally obtained results presented from different studies. Comparisons between the modeling predictions from the present work and other characterization efforts in the literature are shown in Figure 12. For example, Lusti and Gusev [3] predicted an increase in the Young’s modulus by a factor of 6–8 for a CNT reinforced epoxy nanocomposite using a finite element based procedure at a volume fraction of 10%. At a volume fraction of 5%, the improvement in the Young’s modulus is predicted to be between a factor of 2 and 4 (100% increase to 300% increase) which is relatively higher than the predictions made in the present study for $E_{1}$ and $E_{2}$ for the randomly oriented cases at 5% CNT volume fraction of 76.57% and 74.63% increase, respectively. From Figure 12, the upper bound of the modeling prediction (μ+σ) matches closely with the lower bound of the % increase in Lusti and Gusev [3] at 5% CNT volume fraction. Lee et al. [49] investigated the mechanical reinforcement of multi-walled CNTs/polystyrene nanocomposites through nanoindentation and found that the Young’s modulus increased by approximately 90% to 200% with CNT weight concentrations of 5% to 10% (volume fractions of 4.6% to 9.3%), which is close to the predicted values from the present study at 5% CNT weight fraction. In fact, from Figure 12 it can be observed that the values from Lee et al. [49] produce a linear increase in $E_{1}$ that matches well with the initial slope of the $E_{1}$ versus CNT volume fraction from model predictions. Koval’chuk et al. [50] obtained an increase in the Young’s modulus of multi-walled CNTs/polypropylene composites by 66% at 3.6 wt.% (3.3% volume fraction). At a similar volume fraction (3.52% CNT volume fraction), the present study predicts an improvement of approximately 51% for the randomly oriented case. At this volume fraction, the upper bound (μ+σ) of the modeling prediction matches well with the % increase from Koval’chuk et al. [50]. Velasco et al. [51] obtained an increase in the Young’s modulus of multi-walled CNTs/PMMA composites by 32% at 1.5 wt.% (1.38% volume fraction). At a similar volume fraction (1.52% CNT volume fraction), the present study predicts an improvement of approximately 21% for the randomly oriented case. Similar to the earlier comparison, here, the upper bound (μ+σ) of the modeling prediction matches well with the % increase from Koval’chuk et al. [50]. From these comparisons, it can be concluded that the present model predicts mechanical reinforcement in terms of tensile modulus at approximately similar ranges as other studies found in the literature.

Compared to the tensile modulus comparison, there is not much quantitative data for the Poisson’s ratio of CNT/polymer nanocomposites in the literature in terms of experiments. Sepulveda et al. [52] proposed that the decrease in the Poisson’s ratio for aligned CNT/polymer nanocomposites was a result of the CNTs constraining the shrinking of the nanocomposite and found that $\nu_{12}$ (Poisson’s ratio between the alignment direction and transverse direction) decreases from 0.4 (pristine polymer, PDMS) to 0.05 for 1% CNT reinforcement through tensile tests. With randomly oriented CNTs, the degree to which the CNTs will resist the shrinking of the nanocomposite will increase since the some of the CNTs will be more in alignment with the ${\tilde{x}}_{2}$ direction (which was the transverse direction for the aligned cases). In fact, this resistance to shrinking will increase with increasing misalignment of the CNTs. Thus, the Poisson’s ratio $\nu_{12}$ would be the lowest for the randomly oriented cases followed by the imperfectly aligned cases followed by the aligned cases at a given CNT volume fraction and this is consistent with the modeling predictions in the present study observed in Figure 11c.

### 3.2. Estimates of Initial Electrical Conductivity with Straight CNTs

#### 3.2.1. Effect of Orientation and Studying Electrical Percolation

**Figure 13:**
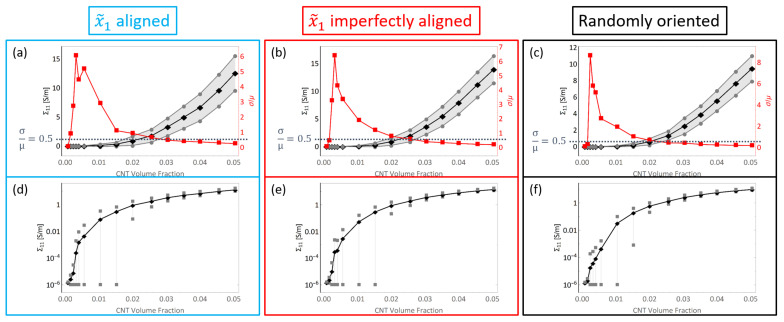
The mean, mean ± standard deviation and coefficient of variation are depicted in black, grey and red, respectively, for $\Sigma_{11}$ for (**a**) straight ${\tilde{x}}_{1}$ aligned CNTs, (**b**) straight ${\tilde{x}}_{1}$ imperfectly aligned ($\pm 15^{\circ }$) CNTs and (**c**) straight randomly oriented cases, respectively. The mean and mean ± standard deviation are depicted in black and grey, respectively, for $\Sigma_{11}$ in semi-log plots for (**d**) straight ${\tilde{x}}_{1}$ aligned, (**e**) straight ${\tilde{x}}_{1}$ imperfectly aligned and (**f**) straight randomly oriented cases, respectively.

The initial electrical conductivity term $\Sigma_{11}$ estimate using a barrier potential of $\lambda =10^{-6}\;\mathrm{eV}$ is shown in Figure 13. The data is presented with two different types of plots, an estimate plot which was seen in the discussion regarding the mechanical stiffness estimates, and a semi-log plot. The estimates are shown for all CNT volume fractions considered in this study and for all three different orientation conditions with straight CNTs—${\tilde{x}}_{1}$ aligned, ${\tilde{x}}_{1}$ imperfectly aligned and randomly oriented. With the increase in CNT volume fraction, conductivity estimate rises for all cases. This is expected since CNT is relatively a much more conductive phase compared to pure epoxy. Furthermore, and more importantly, with the increase in the number of CNTs, i.e., increase in CNT volume fraction, the likelihood of realizing percolation increases significantly. This increased probability can be visualized as increased chances of the fuzzy regions surrounding the CNTs overlapping with one another and thereby creating a network pathway for the current to flow.

It is noteworthy that the increase in $\Sigma_{11}$ is similar for the ${\tilde{x}}_{1}$ aligned and ${\tilde{x}}_{1}$ imperfectly aligned cases. This can be seen in the estimate plots (Figure 13a,b) and semi-log plots (Figure 13d,e) with the mean value reaching as high as 12.54S/m and 13.95S/m for the ${\tilde{x}}_{1}$ aligned and ${\tilde{x}}_{1}$ imperfectly aligned cases, respectively, at 5.04% CNT volume fraction.

The increase in $\Sigma_{11}$ for the randomly oriented case is notably lower than the ${\tilde{x}}_{1}$ aligned or ${\tilde{x}}_{1}$ imperfectly aligned cases with the mean value reaching as high as 9.46S/m obtained at 5.04% CNT volume fraction. The estimates for $\Sigma_{11}$ are lower for the randomly oriented cases due to the effects of alignment since in this case there is an equal probability of forming conductive pathways in the aligned or transverse directions, thereby reducing the total number of paths in the alignment direction.

**Figure 14:**
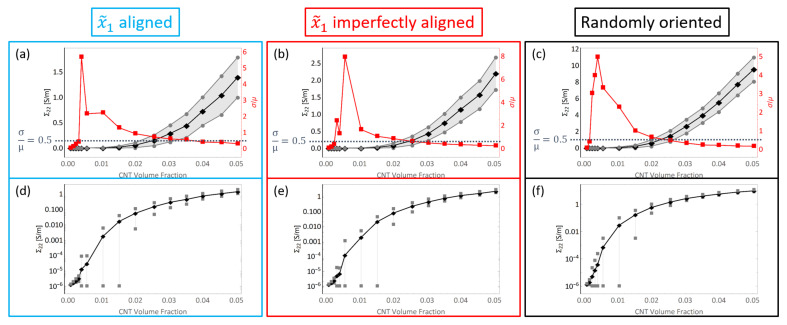
The mean, mean ± standard deviation and coefficient of variation are depicted in black, grey and red, respectively, for $\Sigma_{22}$ for (**a**) straight ${\tilde{x}}_{1}$ aligned CNTs, (**b**) straight ${\tilde{x}}_{1}$ imperfectly aligned ($\pm 15^{\circ }$) CNTs and (**c**) straight randomly oriented cases, respectively. The mean and mean ± standard deviation are depicted in black and grey, respectively, for $\Sigma_{22}$ in semi-log plots for (**d**) straight ${\tilde{x}}_{1}$ aligned, (**e**) straight ${\tilde{x}}_{1}$ imperfectly aligned and (**f**) straight randomly oriented cases, respectively.

In a similar fashion to the plots shown in Figure 13, the initial conductivity term $\Sigma_{22}$ estimates are presented in Figure 14. The increase in $\Sigma_{22}$ is significantly lower for the ${\tilde{x}}_{1}$ aligned and ${\tilde{x}}_{1}$ imperfectly aligned cases with the mean value reaching as high as 1.39S/m and 2.20S/m for the aligned and imperfectly aligned cases, respectively, obtained at 5.04% CNT volume fraction.

The increase in $\Sigma_{22}$ is significantly higher for the randomly oriented cases due to the effects of CNT misalignment since the randomly oriented case has more probability of formation of network conductive paths in the ${\tilde{x}}_{2}$ direction compared to the ${\tilde{x}}_{1}$ aligned and ${\tilde{x}}_{1}$ imperfectly aligned cases. Furthermore, $\Sigma_{22}$ and $\Sigma_{11}$ estimates for the randomly oriented cases are similar since the probability of formation of conductive pathways and the degree of conductivity enhancement of conductive channels is equal in the ${\tilde{x}}_{1}$ and ${\tilde{x}}_{2}$ directions for the randomly oriented case.

From the estimate plots for both $\Sigma_{11}$ and $\Sigma_{22}$, it can be observed that the coefficient of variation σ/μ goes to relatively larger values than compared to the stiffness terms. This means that there exists relatively a much larger degree of deviation in the data sets for the conductivity terms than the stiffness terms. σ/μ is observed to go through a transition from low values observed at the lowest CNT volume fractions, increasing to a peak value at some specific CNT volume fraction before decreasing back down again to smaller values at higher CNT volume fractions for both the $\Sigma_{11}$ and $\Sigma_{22}$ components for all three different orientation conditions. These CNT volume fraction ranges vary depending upon the orientation condition and the conductivity component studied. These values can be found in Table S3. Furthermore, the transitional behavior exhibited by σ/μ indicates that the degree of deviation found in the data for both conductivity terms also transition from low deviations, to high deviation at some intermediate range of volume fractions followed by a shift back down to low deviation values at even higher volume fractions.

This transitional behavior marks the electrical percolation transition region (PTR). This term was introduced in the initial study [8] to represent the CNT volume fraction range where the deviation measured by σ/μ is greater than a threshold value of 0.5. This region exists between two regions of low deviation in the data. In other words, at these CNT volume fractions, a high variation in the effective conductivity is observed due to the stochastic nature of the occurrence of network formation. With lower volume fractions, most if not all of the realizations are unpercolated because of the dearth of CNTs, thereby making the effective conductivity terms for all realizations very low and therefore there is less deviation in the data. With higher volume fractions, most realizations are percolated because of the abundance of CNTs, thereby making the effective conductivity terms for all realizations high and therefore there is less deviation in the data. Furthermore, the PTR marks the range of CNT volume fractions over which the conductivity estimates increase sharply through orders of magnitude, transitioning away from the low conductivity of the polymer ($10^{-6}\;S/m$ order of magnitude) to higher conductivity values (above $10^{1}\;S/m$ order of magnitude) since as an increasing number of effective conductivity values in subsequent data sets (in terms of increasing volume fraction) are a result of percolated realizations.

The semi-log plots are an alternative representation of the data to better observe the increase in the conductivity estimates through orders of magnitude from the conductivity of the pure epoxy of $10^{-6}\;S/m$. Mean values are depicted with black data points. Deviation bars are depicted in grey which show the μ±σ. In these plots, the lower bars are shorter than the upper bars since the logarithmic scale of the effective conductivity axis does not display in a balanced fashion.

The PTR for the ${\tilde{x}}_{1}$ imperfectly aligned case occurs between 0.24% and 2.56% CNT volume fractions for $\Sigma_{11}$ and between 0.32% and 2.56% CNT volume fractions for $\Sigma_{22}$ which can be observed from Figure 13b and Figure 14b. The onset of the PTR for $\Sigma_{11}$ shifts to slightly higher volume fractions for the ${\tilde{x}}_{1}$ imperfectly aligned case (0.24% CNT volume fraction) compared to the ${\tilde{x}}_{1}$ aligned case (0.16% CNT volume fraction) due to the slight degree of misalignment in the ${\tilde{x}}_{1}$ imperfectly aligned case decreasing the probability of formation of percolated pathways in the alignment direction. Misalignment is also the reason why the onset of the PTR for $\Sigma_{22}$ is shifted to slightly lower volume fractions for the ${\tilde{x}}_{1}$ imperfectly aligned case (0.32% CNT volume fraction) compared to the ${\tilde{x}}_{1}$ aligned case (0.40% CNT volume fraction) since the probability of formation of percolated pathways in the transverse direction increases with misalignment. Additionally, the transition out of the PTR for $\Sigma_{22}$ also happens at lower CNT volume fractions for the ${\tilde{x}}_{1}$ imperfectly aligned case (2.56%) than the ${\tilde{x}}_{1}$ aligned case (3.52%) since the transition from a majority unpercolated data set to a majority percolated data set in the transverse direction happens at lower CNT volume fractions when misalignment is present.

**Figure 15:**
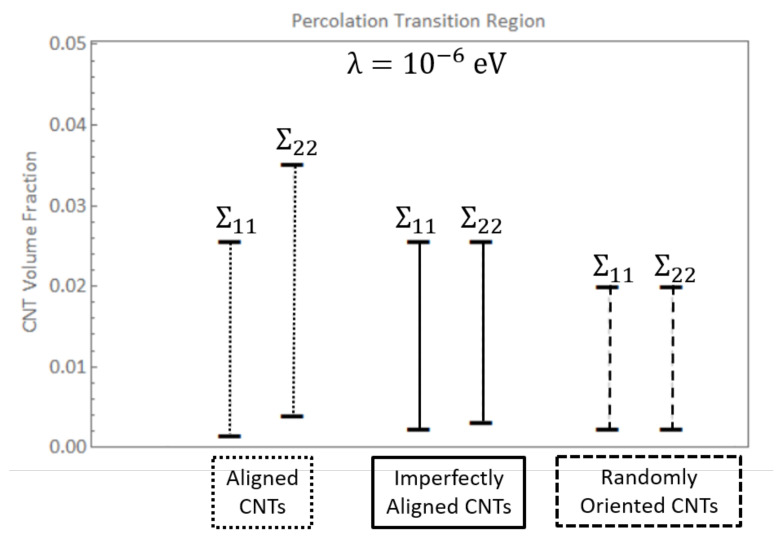
Percolation transition region shown for $\Sigma_{11}$ and $\Sigma_{22}$ for straight ${\tilde{x}}_{1}$ aligned, straight ${\tilde{x}}_{1}$ imperfectly aligned and straight randomly oriented cases.

The PTR for the randomly oriented case is found to be between 0.24% and 2.00% CNT volume fractions for both $\Sigma_{11}$ and $\Sigma_{22}$ which can be observed from Figure 13c and Figure 14c. The PTR is exactly the same for $\Sigma_{11}$ and $\Sigma_{22}$ since the orientation of CNTs is not biased in any direction for the randomly oriented case. The transition out of the PTR occurs at lower CNT volume fractions for both $\Sigma_{11}$ and $\Sigma_{22}$ for the randomly oriented case than for the PTR corresponding to $\Sigma_{11}$ or $\Sigma_{22}$ for ${\tilde{x}}_{1}$ aligned or ${\tilde{x}}_{1}$ imperfectly aligned cases. This indicates that the deviation in the data sets decrease at a faster rate with increase in CNT volume fraction for the randomly oriented case than the ${\tilde{x}}_{1}$ aligned or ${\tilde{x}}_{1}$ imperfectly aligned cases. The PTR for both $\Sigma_{11}$ and $\Sigma_{22}$ for the three different orientation conditions are summarized in Figure 15.

**Figure 16:**
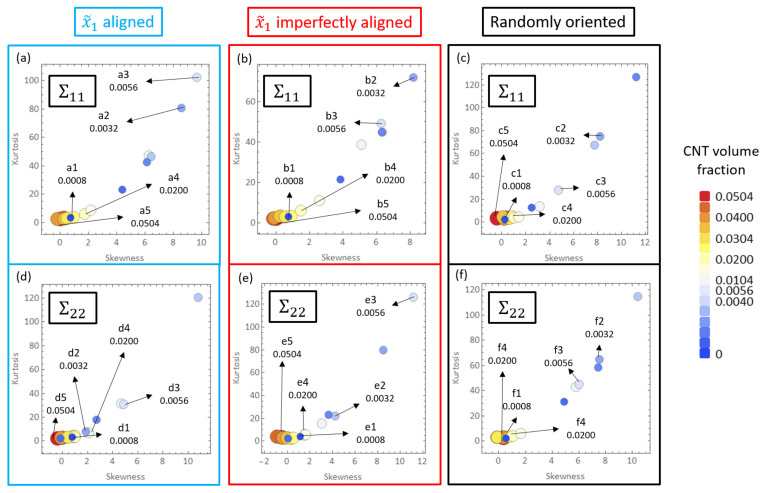
Skewness-Kurtosis plots for $\Sigma_{11}$ for the (**a**) straight ${\tilde{x}}_{1}$ aligned case, (**b**) straight ${\tilde{x}}_{1}$ imperfectly aligned case and (**c**) straight randomly oriented case and for $\Sigma_{22}$ for the (**d**) straight ${\tilde{x}}_{1}$ aligned case, (**e**) straight ${\tilde{x}}_{1}$ imperfectly aligned case and (**f**) straight randomly oriented.

The deviation found in the estimates for $\Sigma_{11}$ and $\Sigma_{22}$ for all three different orientation conditions can be analyzed with the help of skewness-kurtosis plots which are presented in Figure 16. Additionally, the skewness and kurtosis values for all cases are shown in Tables S4 and S5 with the CNT volume fractions corresponding to the PTR being marked with thick lined boxes. Skewness measures asymmetry in a data set about the mean value. Typically for a unimodal distribution, a negative skew is indicative a longer tailed left hand side and a positive skew is indicative of a longer tailed right hand side. Kurtosis measures the tailedness of the distribution, i.e., it is indicative of how significant the extremities of the distribution are as opposed the data near the mean. A normal distribution exhibits skewness and kurtosis values of 0 and 3, respectively. For all three orientation conditions, it can be seen that the skewness and kurtosis measures transition from low values at low CNT volume fraction (i.e., close to values that are typical of a normal distribution), to relative larger values for an intermediate range of volume fraction, followed by a downward trend at higher volume fractions. The volume fractions over which this transition occurs differs depending upon the orientation case analyzed and the conductivity component studied ($\Sigma_{11}$ vs. $\Sigma_{22}$).

The shift in the PTR observed for the transverse direction compared to the aligned direction can be visualized with the skewness-kurtosis plots. In both Figure 16a,b (corresponding to $\Sigma_{11}$ skewness-kurtosis plots for the ${\tilde{x}}_{1}$ aligned and ${\tilde{x}}_{1}$ imperfectly aligned cases, respectively), the skewness-kurtosis values start to increase almost immediately with high values observed starting from 0.16% CNT volume fraction. However, in both Figure 16d,e (corresponding to $\Sigma_{22}$ skewness-kurtosis plots for the ${\tilde{x}}_{1}$ aligned and ${\tilde{x}}_{1}$ imperfectly aligned cases, respectively), it is seen that the four smallest volume fractions simulated have relatively lower values of skewness-kurtosis indicating that the deviation starts to increase significantly at relatively higher CNT volume fractions compared to the corresponding cases for $\Sigma_{11}$. This trend can be corroborated with the values in Tables S4 and S5.

For the randomly oriented case, the transition behavior in skewness-kurtosis values is roughly the same for both $\Sigma_{11}$ and $\Sigma_{22}$, with the range of CNT volume fractions exhibiting high values of skewness-kurtosis being 0.16% to 2.00% (taking threshold skewness and kurtosis values of 1 and 3.5, respectively) for both conductivity components. This observation is consistent with the PTR being the same range for both conductivity components for the randomly oriented case and in fact the range of 0.16% to 2.00% overlaps almost entirely with the PTR (0.24% to 2.00%).

The data points found in Figure 16 are positive in their skewness values. This is indicative of right-tailedness of the distributions. This occurs due to the steady increase in the number of high effective conductivity realizations with increase in CNT volume fraction, i.e., when CNT volume fraction is low, percolated realizations are rare thereby making the distributions extremely skewed and right-tailed. However, as the distributions become dominated with percolated realizations with increased volume fraction, the tailedness decreases.

**Figure 17:**
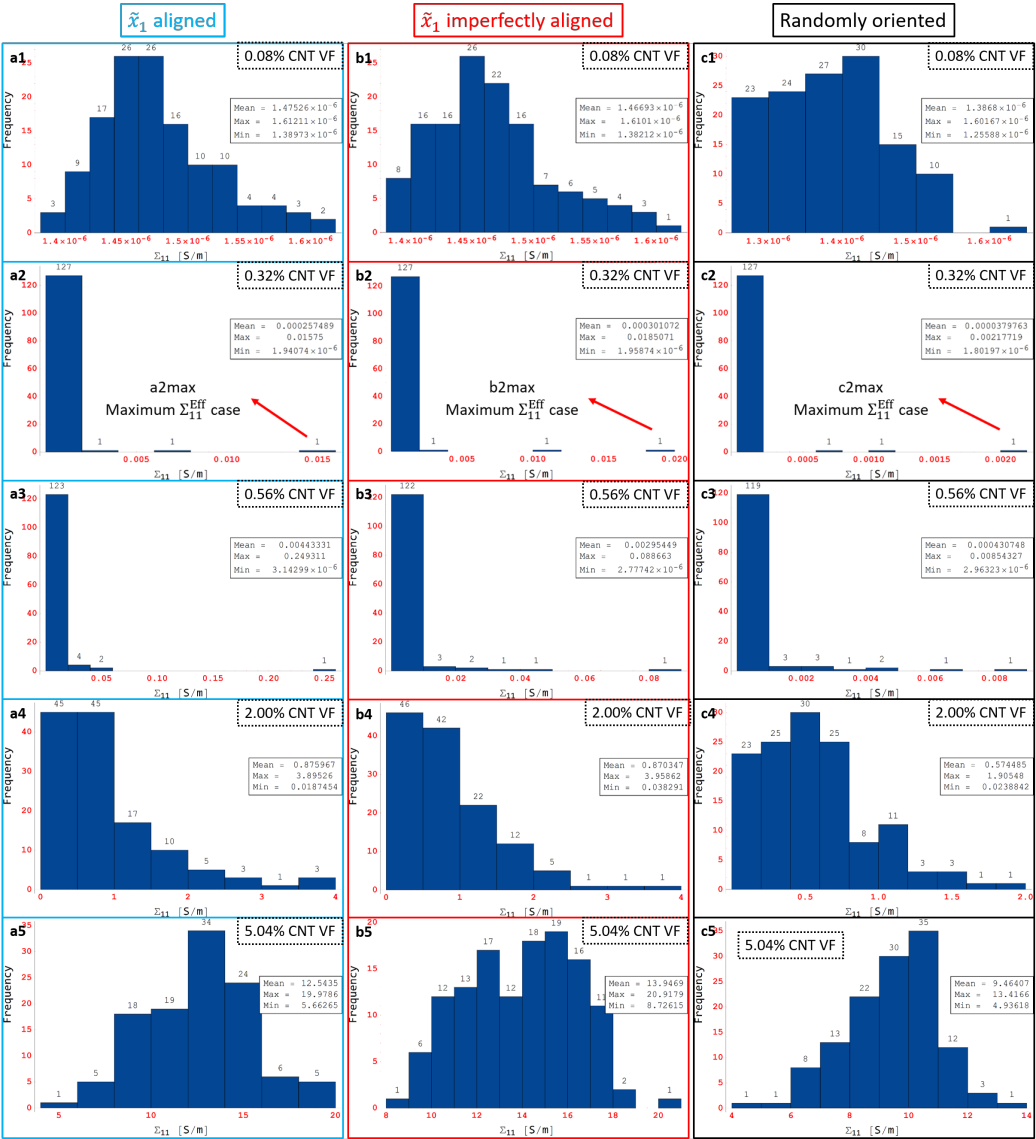
Histograms for $\Sigma_{11}$ at 0.08%, 0.32%, 0.56%, 2.00% and 5.04% CNT volume fractions for all orientation conditions.

Some of the data shown in Figure 16a–c are marked as a1 through a5, b1 through b5 and c1 through c5, respectively, which correspond to CNT volume fractions 0.08%, 0.32%, 0.56%, 2.00% and 5.04%, respectively. The histogram distributions of the effective conductivities $\Sigma_{11}^{Eff}$ are presented in Figure 17 at the volume fractions corresponding to a1 through a5, b1 through b5 and c1 through c5. Figure 17a1,b1,c1 show distributions that are close to a normal distribution which correlates well with the relatively low skewness and kurtosis values observed for a1, b1 and c1 in Figure 16a–c, i.e., none of the realizations in any of the data sets corresponding to a1, b1 or c1 have led to percolation since the presence of only 1 CNT (0.08% CNT volume fraction) cannot lead to percolation in either the ${\tilde{x}}_{1}$ or the ${\tilde{x}}_{2}$ direction for any orientation condition.

Figure 17a2,b2,c2 are markedly different from the normal distribution type response. A heavily right-skewed response is observed since some of the realizations in the corresponding data sets give rise to relatively high levels of effective conductivity $\Sigma_{11}^{Eff}$. These high conductivity realizations represent the percolated data points and are a minority in the data set where most realizations are still largely unpercolated and therefore low conductivity. These highly skewed distributions correlate well with the corresponding skewness and kurtosis values observed in Figure 16a–c for a2, b2 and c2, respectively. Figure 17a3,b3,c3 exhibit the same type of response as a2, b2 and c2 with highly right-skewed distributions that correlate well with the corresponding skewness and kurtosis values in Figure 16a–c. The CNT volume fraction corresponding to a3, b3 and c3 is 0.56%, which is still well within the PTR for all three orientation conditions meaning that the percolated data points are still a minority in the corresponding data sets where most realizations are still largely unpercolated.

Figure 17a4,b4,c4 finally show a return of the distributions towards more of a normal distribution type behavior since the fraction of percolated data points in the corresponding data sets has increased significantly. Cases a4 and b4 appear to be more heavily right skewed than c4 since the maximum effective conductivity $\Sigma_{11}^{Eff}$ cases are much larger for the ${\tilde{x}}_{1}$ aligned and ${\tilde{x}}_{1}$ imperfectly aligned cases than for the randomly oriented cases due to the effect of alignment. The slightly less skewed distribution observed in Figure 17c4 signals the end of the PTR for $\Sigma_{11}$ for the randomly oriented cases since this means that increasingly more number of realizations are percolated and therefore the statistical variation in the data is reducing as the volume fraction increases. The increasingly normal distribution type response correlates well with the lower skewness-kurtosis values observed in Figure 16a–c for a4, b4 and c4, respectively. Figure 17a5,b5,c5 show normal distribution type responses since all the data points in the corresponding data sets correspond to percolated realizations in the ${\tilde{x}}_{1}$ direction, i.e., the PTR has been completed in terms of volume fraction for all three different orientation conditions. The entire transition of $\Sigma_{11}^{Eff}$ distributions can be correlated well with a1 through a5, b1 through b5 and c1 through c5 for $\Sigma_{11}$ for ${\tilde{x}}_{1}$ aligned, ${\tilde{x}}_{1}$ imperfectly aligned and randomly oriented cases, respectively, using the histogram distributions in Figure 17 and the skewness-kurtosis plots in Figure 16a–c.

**Figure 18:**
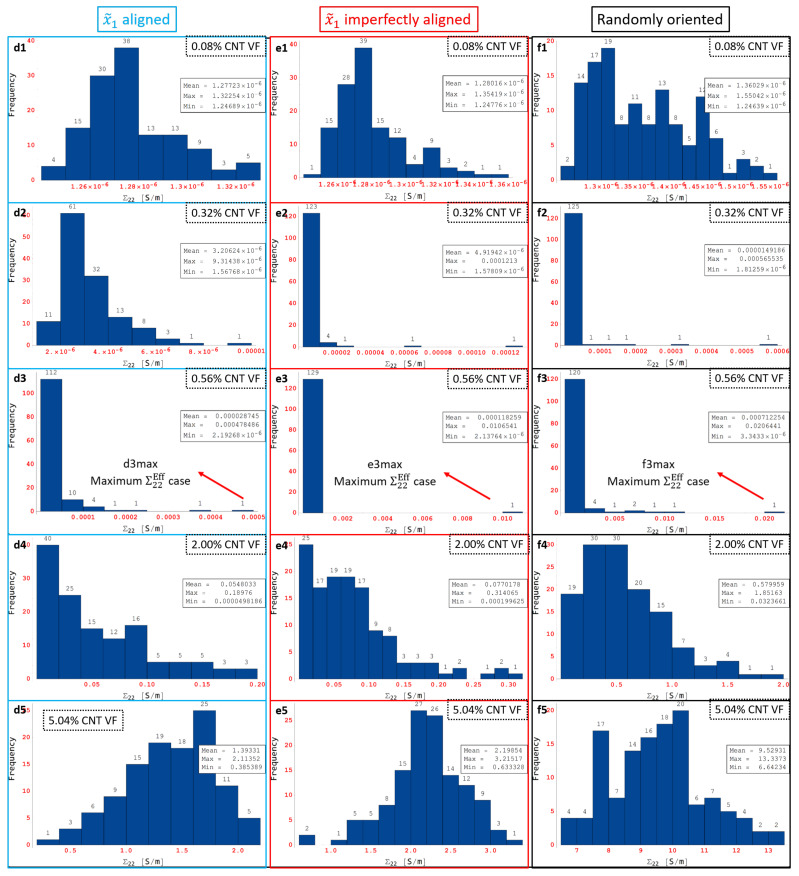
Histograms for $\Sigma_{22}$ at 0.08%, 0.32%, 0.56%, 2.00% and 5.04% CNT volume fractions for all orientation conditions.

Some of the data shown in Figure 16d–f are marked as d1 through d5, e1 through e5 and f1 through f5, respectively, which correspond to CNT volume fractions 0.08%, 0.32%, 0.56%, 2.00% and 5.04%, respectively. The histogram distributions of the effective conductivities $\Sigma_{22}^{Eff}$ are presented in Figure 18 at the volume fractions corresponding to d1 through d5, e1 through e5 and f1 through f5. Similar to the $\Sigma_{11}$ conductivity component, the entire transition of $\Sigma_{22}^{Eff}$ distributions can be correlated well with d1 through d5, e1 through e5 and f1 through f5 for $\Sigma_{22}$ for ${\tilde{x}}_{1}$ aligned, ${\tilde{x}}_{1}$ imperfectly aligned and randomly oriented cases, respectively, using the histogram distributions in Figure 18 and the skewness-kurtosis plots in Figure 16d–f. The histogram distributions corresponding to f1 through f5 are very similar to c1 through c5 since the randomly oriented case is unbiased with respect to direction of analysis. The onset of the PTR at lower CNT volume fractions for the ${\tilde{x}}_{1}$ imperfectly aligned and randomly oriented cases compared to the ${\tilde{x}}_{1}$ aligned case can be observed through Figure 18d2,e2,f2, where case d2 exhibits more of a normal distribution type response since $\Sigma_{22}$ for the ${\tilde{x}}_{1}$ aligned case is yet to enter the PTR at the corresponding volume fraction of 0.32%, but cases e2 and f2 exhibit right skewed distributions indicating that the PTR has started for both the ${\tilde{x}}_{1}$ imperfectly aligned and randomly oriented cases.

**Figure 19:**
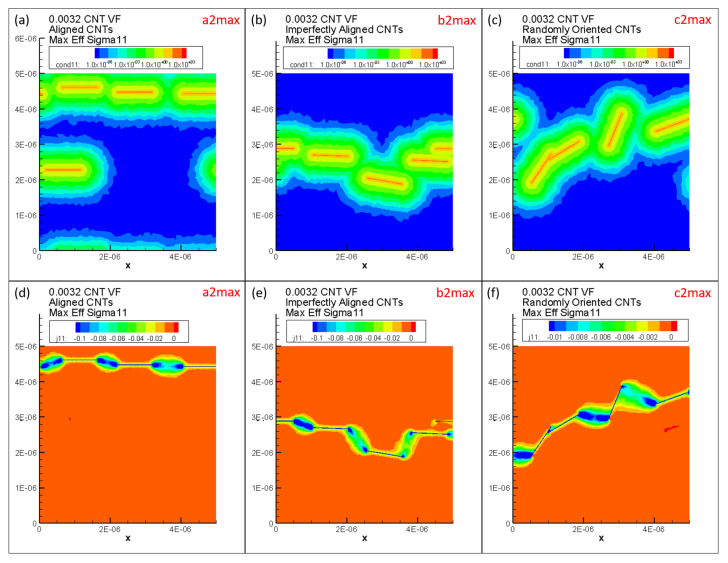
Local conductivity $\Sigma_{11}\;S/m$ for the maximum $\Sigma_{11}^{Eff}$ realizations at 0.32% CNT volume fraction for (**a**) ${\tilde{x}}_{1}$ aligned, (**b**) ${\tilde{x}}_{1}$ imperfectly aligned and (**c**) randomly oriented cases with local current density $J_{1}\;A/m^{2}$ for the maximum $\Sigma_{11}^{Eff}$ realizations at 0.32% CNT volume fraction for (**d**) ${\tilde{x}}_{1}$ aligned, (**e**) ${\tilde{x}}_{1}$ imperfectly aligned and (**f**) randomly oriented cases.

Maximum $\Sigma_{11}^{Eff}$ is marked as a2max, b2max and c2max in Figure 17a2,b2,c2, respectively, for the ${\tilde{x}}_{1}$ aligned, ${\tilde{x}}_{1}$ imperfectly aligned and randomly oriented cases, respectively. Each of a2max, b2max and c2max make up the last bin in their corresponding histogram distributions and correspond to values of $\Sigma_{11}^{Eff}=0.0158\;S/m$, $\Sigma_{11}^{Eff}=0.0185\;S/m$ and $\Sigma_{11}^{Eff}=0.0022\;S/m$, respectively. In order to illustrate the large amounts of deviation exhibited by the conductivity data sets for $\Sigma_{11}$, these maximum effective conductivity cases of a2max, b2max and c2max are investigated further with the contour plots showing local conductivity $\Sigma_{11}\;S/m$ and local current density $J_{1}\;A/m^{2}$ presented in Figure 19 for each of the cases. Since the contours in Figure 19 are under electrical boundary conditions corresponding with test 1 (measuring $\Sigma_{11}$), the primary direction of current flow is across the width of the realizations. Due to the dispersion of CNTs occurring in a favorable manner, the clouds of enhanced conductivity overlap with one another creating network pathways in the ${\tilde{x}}_{1}$ direction thereby making the realization percolated. This can be confirmed from the contours showing local current density $J_{1}\;A/m^{2}$ which clearly show current flow in the ${\tilde{x}}_{1}$ direction as a result of the conductive networks formed in all three cases—a2max, b2max and c2max. The resulting effective conductivity $\Sigma_{11}^{Eff}$ value for c2max is an order of magnitude lower than that for a2 max and b2max which can be correlated with the conductivity contours since the tunneling clouds narrowly overlap one another for c2max thereby creating a weak conductive network as opposed to the strong conductive networks seen in a2max and b2max which show extensive overlap for their tunneling clouds.

Thus, the effective conductivity measures depend upon (i) CNT dispersion controlling the probability of formation of percolated networks which is also influenced by the orientation of CNTs and (ii) CNT orientation controlling the nature of the conductive pathway (direct networks vs. meandering networks). Since all other realizations result in much lower values of $\Sigma_{11}^{Eff}$ in their corresponding orientation case, it is clear that the probability of percolation is low at this CNT volume fraction for all three orientation conditions, i.e., outside of a2max, b2max and c2max cases, the remaining cases at this volume fraction have unfavorable CNT dispersions and selections of orientation angles. Thus, the majority of the data sets corresponding to a2, b2 and c2 consist of unpercolated data points with a few percolated data points resulting in a heavily right skewed distribution which can be observed in Figure 17a2,b2,c2. A similar exercise is done to analyze the large deviation in the individual $\Sigma_{22}^{Eff}$ values at 0.56% CNT volume fraction by investigating the maximum $\Sigma_{22}^{Eff}$ cases in the SI.

**Figure 20:**
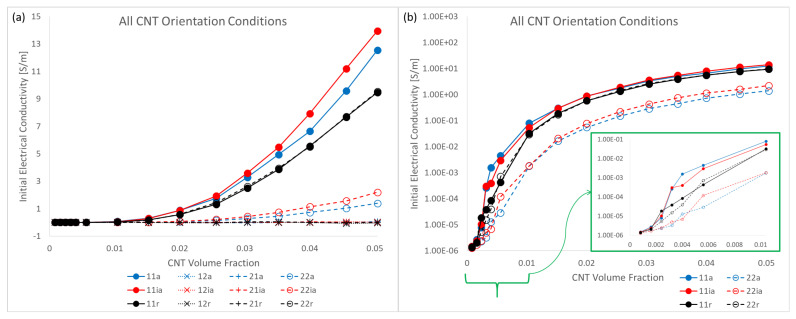
(**a**) Mean of all initial electrical conductivity terms $\Sigma_{ij}$ for all three orientation conditions and (**b**) mean of only $\Sigma_{11}$ and $\Sigma_{22}$ on a semi-log scale for all three orientation conditions. (Inset in **b**) shows mean of $\Sigma_{11}$ and $\Sigma_{22}$ for volume fractions ≤ 1.04%).

Estimates for conductivity components $\Sigma_{12}$ and $\Sigma_{21}$ were found to be near zero values for the ${\tilde{x}}_{1}$ aligned case in the initial study [8]. Similarly, these components are also found to be near zero values for the ${\tilde{x}}_{1}$ imperfectly aligned and randomly oriented cases where individual effective values $\Sigma_{12}^{Eff}$ and $\Sigma_{21}^{Eff}$ are very small compared to the other effective components $\Sigma_{11}^{Eff}$ and $\Sigma_{22}^{Eff}$ and (ii) the estimates for $\Sigma_{12}$ and $\Sigma_{21}$ when averaged result in an insignificantly small value for all CNT volume fractions due to an approximately symmetric distribution of the data with half the data points being marginally positive and the other half being marginally negative. Therefore, estimates for these components are assumed as zero for all CNT volume fractions for all orientation conditions. The estimate of every component of the initial electrical conductivity tensor $\Sigma_{ij}$ for all orientation conditions is presented in Figure 20a, which demonstrates that the conductivity estimates for $\Sigma_{12}$ and $\Sigma_{21}$ are near zero values and are negligible compared to $\Sigma_{11}$ and $\Sigma_{22}$ and thus can be treated as zero. A semi-log plot showing the conductivity components $\Sigma_{11}$ and $\Sigma_{22}$ for all orientation conditions is presented in Figure 20b in order to visualize the increase in conductivity estimates over orders of magnitude, especially at lower CNT volume fractions. From Figure 20 it is observed that $\Sigma_{11}$ is consistently larger for the ${\tilde{x}}_{1}$ aligned and ${\tilde{x}}_{1}$ imperfectly aligned cases compared to the randomly oriented case which is expected since (i) there is more probability of percolation in the ${\tilde{x}}_{1}$ direction with alignment in the ${\tilde{x}}_{1}$ direction and (ii) percolated realizations have relatively more direct pathways for electrical conduction as opposed to the randomly oriented case. $\Sigma_{22}$ is consistently larger for the randomly oriented case than the ${\tilde{x}}_{1}$ aligned and ${\tilde{x}}_{1}$ imperfectly aligned cases for similar reasons. $\Sigma_{11}$ and $\Sigma_{22}$ for the randomly oriented case are observed to be almost identical in both Figure 20a and Figure 20b corroborating the unbiased orientation of CNTs in the randomly oriented case. For $\Sigma_{11}$, the ${\tilde{x}}_{1}$ aligned and ${\tilde{x}}_{1}$ imperfectly aligned cases surpass one another at different CNT volume fractions in the range of CNT volume fractions corresponding to percolation transition for these cases ([0.16% to 2.56%] for ${\tilde{x}}_{1}$ aligned and [0.24% to 2.56%] for ${\tilde{x}}_{1}$ imperfectly aligned). However, starting from the end of the PTR for both ${\tilde{x}}_{1}$ aligned and ${\tilde{x}}_{1}$ imperfectly aligned cases (at 2.56% CNT volume fraction for both cases), the ${\tilde{x}}_{1}$ imperfectly aligned estimate is consistently larger than the ${\tilde{x}}_{1}$ aligned estimate for $\Sigma_{11}$. This indicates that once the data sets for estimating $\Sigma_{11}$ transition to majority percolated data sets after the PTR, the ${\tilde{x}}_{1}$ imperfectly aligned case offers a larger number of conductive pathways as a result of the slight misalignment. In contrast, $\Sigma_{22}$ for the ${\tilde{x}}_{1}$ imperfectly aligned case yields estimates that are consistently larger than that for the ${\tilde{x}}_{1}$ aligned case due to the effects of misalignment.

#### 3.2.2. Comparison to Other Studies in the Literature

Several other studies have explored and analyzed electrical percolation and the conductivity increase of CNT embedded polymers. There is limited data available on aligned or imperfectly aligned conductivity enhancements in the literature. The conductivity estimates and percolation values for the aligned case matched will with a few experimental studies compared to in the initial study [8]. Since these experimental studies likely exhibit some degree of misalignment as it is not possible to manufacture CNT/polymer nanocomposites with perfect alignment of CNTs, the imperfectly aligned cases can also be compared to these studies. The conductivity estimates and percolation values for aligned and imperfectly aligned in the present study match very closely, and therefore the experimental comparison in the initial study [8] can be extended to the imperfectly aligned cases. However, in the present study the model predicts that $\Sigma_{11}$ is slightly greater for the imperfectly aligned case compared to the aligned case for the range of CNT volume fractions post percolation. A similar observation was made by Behnam et al., who showed that CNT/polymer films with partially aligned CNTs exhibit lower resistivities than films with perfectly aligned CNTs.

**Figure 21:**
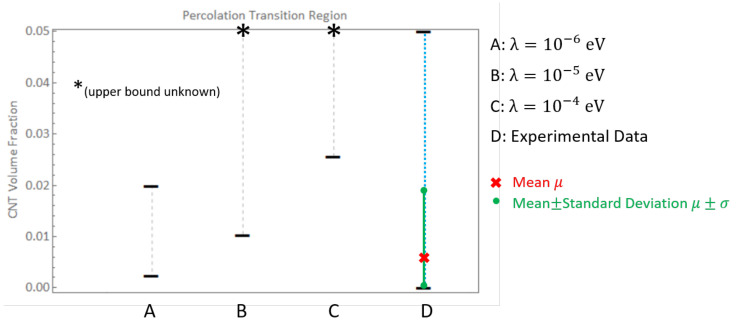
Percolation transition region depicted for $\Sigma_{11}=\Sigma_{22}$ for straight randomly oriented cases for 3 different barrier potentials; CNT volume fraction range depicted by D from review study [7].

In the present work, comparisons are done primarily for the randomly oriented case since most characterization efforts study randomly oriented CNTs. Bauhofer and Kovacs [7] assembled a large data set of experimental parameters of numerous publications and found that for 23 different publications that studied CNT/epoxy nanocomposites using either single-walled or multi-walled CNT reinforcement, $\phi_{c}$ or percolation threshold was found to be between 0.0021 wt.% and 5 wt.%, which is consistent with the predictions in the present study for $\lambda =10^{-6}\;\mathrm{eV}$ for randomly oriented CNTs where the PTR was found to be between 0.26 wt.% and 2.17 wt.% ([0.24%, 2.00%] in terms of CNT volume fraction). This is observed in Figure 21 which presents the PTR for three different barrier potential values of $\lambda =10^{-6}\;\mathrm{eV}$, $\lambda =10^{-5}\;\mathrm{eV}$ and $\lambda =10^{-4}\;\mathrm{eV}$ for the randomly oriented cases along with the mean μ and mean ± standard deviation μ±σ of the percolation thresholds reported from 23 different epoxy/CNT nanocomposite experimental studies [7]. (The μ−σ point is capped at a volume fraction of 0 since a negative volume fraction is physically impractical. The PTRs for $\Sigma_{11}$ and $\Sigma_{22}$ are exactly the same for each barrier potential for the randomly oriented cases and hence only one range is shown unlike Figure 15). It is clearly observed that experimental evidence matches well with the PTR reported for $\lambda =10^{-6}\;\mathrm{eV}$. Lower values of barrier potential resulted in hopping range of nanometers which would not be percolated at these low volume fractions as there would little to no tunneling overlap.

Bauhofer and Kovacs [7] also comment that for $\phi_{c}>$ 0.2 wt.%, there were no more than two studies that they could find for any polymer matrix, which supported their supposition that optimized dispersion methods could lead to percolation being attained at $\phi_{c}∼$ 0.1 wt.% for nearly any CNT/polymer system. This indicates that the 2 cases with $\phi_{c}>$ 0.2 wt.% were most likely a result of poor dispersions and better optimized dispersions resulted in percolation with an upper bound of 0.2 wt.%. These values are consistent with the data obtained using $\lambda =10^{-6}\;\mathrm{eV}$ and thus lower values of λ, i.e., larger tunneling distances, are not needed.

The electrical conductivity of the CNT/epoxy systems was found to be orders of magnitude higher than that of epoxy for all studies included in the data set assembled together by Bauhofer and Kovacs [7]. However, the conductivity enhancement reported by these studies varied by orders of magnitude with some studies reporting low conductivity enhancement with low maximum conductivity values, for example, $2\times 10^{-5}\;S/m$ at 20 wt.% [53], $1\times 10^{-5}\;S/m$ at 8 wt.% [54], $5\times 10^{-3}\;S/m$ at 10 wt.% [55] etc. and some other studies reporting much larger degrees of conductivity enhancement with higher maximum conductivity values, for example, 2S/m at 1 wt.% [56], 5S/m at 3 wt.% [57], 1S/m at 2 wt.% [58] etc. The modeling predictions in the present study with regards to conductivity enhancement for the randomly oriented case using $\lambda =10^{-6}\;\mathrm{eV}$ are more consistent with the higher end of the reported values in these data sets, with the conductivity enhancement increasing non-linearly between values of ∼0.2S/m and ∼9.5S/m between 1.52% and 5.04% CNT volume fractions (corresponding to a range of ∼1.65 wt.% to ∼5.45 wt.%). Thus, in addition to inducing the right range of percolation concentration values, $\lambda =10^{-6}\;\mathrm{eV}$ also produces the right value of conductivity matching well with experimental results.

### 3.3. Estimates of Nanocomposite Piezoresistivity with Straight CNTs

#### 3.3.1. Effect of Orientation and Percolation Transition Region

**Figure 22:**
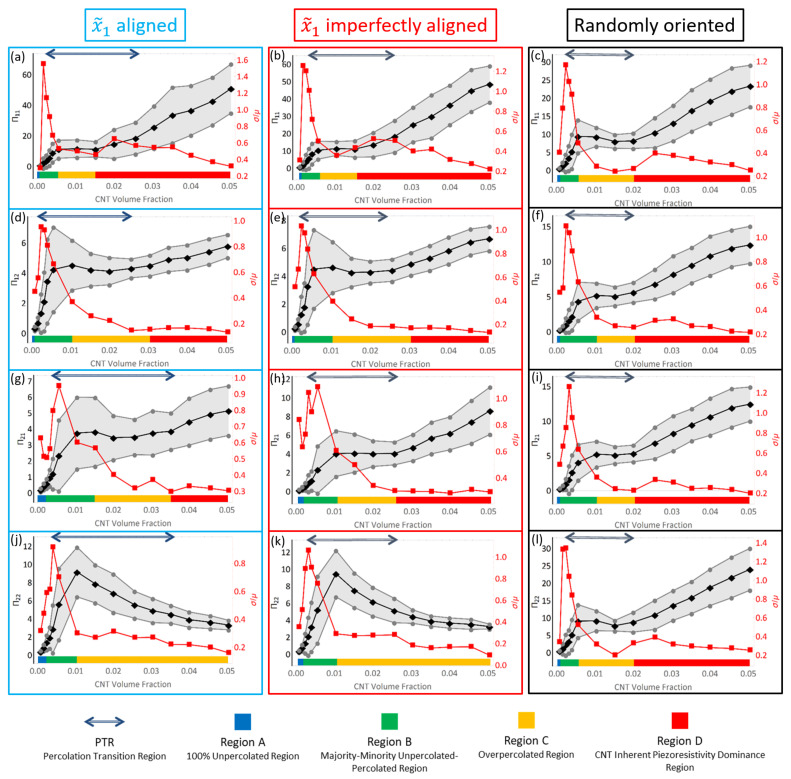
The mean, mean ± standard deviation and coefficient of variation shown in black, grey and red, respectively, for $\Pi_{11}$, $\Pi_{12}$, $\Pi_{21}$ and $\Pi_{22}$ for ${\tilde{x}}_{1}$ aligned, ${\tilde{x}}_{1}$ imperfectly aligned and randomly oriented cases. $\Pi_{11}$, $\Pi_{12}$, $\Pi_{21}$ and $\Pi_{22}$ correspond to the first row (**a**–**c**), second row (**d**–**f**), third row (**g**–**i**) and fourth row (**j**–**l**), respectively. Regions A, B, C and D along with the PTR are denoted in each plot.

Figure 22 presents the estimates for piezoresistivity terms $\Pi_{11}$, $\Pi_{12}$, $\Pi_{21}$ and $\Pi_{22}$ for all three different orientation conditions of ${\tilde{x}}_{1}$ aligned, ${\tilde{x}}_{1}$ imperfectly aligned and randomly oriented. It is interesting to note that for all three orientation conditions, the off diagonal components $\Pi_{12}$ and $\Pi_{21}$ are of significant value. This suggests that there is an observable and non-trivial sensing effect observed in the direction that is perpendicular to that of the loading, i.e., the term $\Pi_{12}$ measures resistivity change in ${\tilde{x}}_{1}$ when mechanical loading is done in ${\tilde{x}}_{2}$ and vice versa for $\Pi_{21}$. For the ${\tilde{x}}_{1}$ aligned and ${\tilde{x}}_{1}$ imperfectly aligned cases, $\Pi_{11}$ goes to large values of 50.89 and 48.55, respectively, at 5.04% CNT volume fraction, which are higher than all other piezoresistivity terms in all orientation conditions. $\Pi_{22}$ is significantly lower than $\Pi_{11}$ in the ${\tilde{x}}_{1}$ aligned and ${\tilde{x}}_{1}$ imperfectly aligned cases, reaching maximum values of 7.85 and 9.47 at 1.52% CNT volume fraction and 1.04% CNT volume fraction for the ${\tilde{x}}_{1}$ aligned and ${\tilde{x}}_{1}$ imperfectly aligned cases, respectively. In contrast, the $\Pi_{11}$ and $\Pi_{22}$ response for the randomly oriented case are similar to one another, with the maximum $\Pi_{11}$ and $\Pi_{22}$ values found to be 23.35 and 23.99, respectively, both occurring at 5.04% CNT volume fraction. All piezoresistivity terms presented in Figure 22 show complex behavior of growth, followed by a local maximum, a plateau region and, with the exception of $\Pi_{22}$ for the ${\tilde{x}}_{1}$ aligned and ${\tilde{x}}_{1}$ imperfectly aligned case, a second region of increase with increasing CNT volume fraction. It is noteworthy that this response is dissimilar to the monotonic increase in the effective estimate that was found for the initial conductivity and stiffness terms (Figure 7 and Figure 20, respectively).

The coefficient of variation σ/μ approximately follows a similar response like the response observed for the conductivity components (Figure 20), with an initial increasing portion, followed by a maximum and then a steady decline to low values. It should be noted that σ/μ for the piezoresistivity components do not reach the same levels as that of the conductivity components, with the maximum values in Figure 22 being closer to 1 to 1.5 rather than 5 to 6. This occurs because the relative difference between the maximum and minimum measures of effective piezoresistivity ($\Pi_{11}^{Eff}$, $\Pi_{12}^{Eff}$, $\Pi_{21}^{Eff}$ or $\Pi_{22}^{Eff}$) is at the maximum 1 or 2 orders of magnitude, where as the relative difference between unpercolated and percolated realizations corresponding to maximum and minimum effective conductivity ($\Sigma_{11}^{Eff}$ or $\Sigma_{22}^{Eff}$) can be several orders of magnitude depending on the extent of percolation effects.

Figure 22 shows the PTR for $\Sigma_{11}$ derived from the threshold measure of 0.5 for the plots showing $\Pi_{11}$ and $\Pi_{12}$. This is done because these piezoresistivity terms measure the relative change in resistivity in the direction of alignment. On the other hand, for the plots showing $\Pi_{21}$ and $\Pi_{22}$, the PTR for $\Sigma_{22}$ is shown, which is done since these piezoresistivity terms measure the relative change in resistivity in the direction perpendicular to the alignment direction. Comparisons between the PTR and the progression of the estimates shown in black, it can be seen that the start of the PTR is typically accompanied with the plateauing of the curve. The extent of this plateauing effect varies between the different piezoresistive terms. The start of the PTR also appears to correspond with a local peak in the piezoresistive component estimate. To better understand these complex responses for the aligned case, the initial study [8] showed different regions corresponding with a specific color legend on each of the plots. The same approach is employed here to analyze the responses for all three different orientation conditions. The regions are (i) Region A: marks the region before the start of the PTR. It shows low deviation in the data (ii) Region B: typically starts with the onset of the PTR and exhibits higher amounts of deviation (iii) Region C: corresponds with the flattening of the curve (iv) Region D: a region of monotonic increase at high volume fractions. A more detailed discussion regarding each region is carried out in the SI.

**Figure 23:**
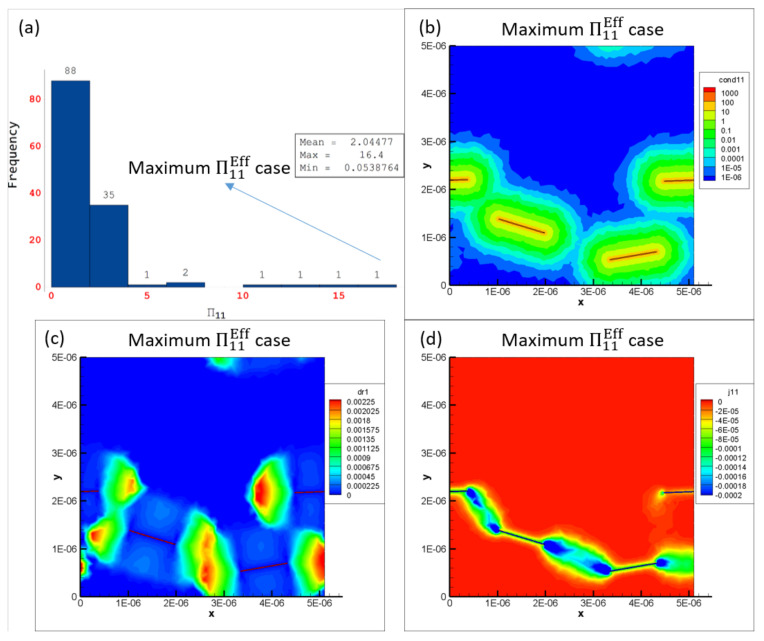
(**a**) Histogram for $\Pi_{11}$ at 0.24% CNT volume fraction for the randomly oriented case with (**b**) local conductivity $\Sigma_{11}\;S/m$, (**c**) relative change in resistivity $\Delta \rho_{1}/\rho_{1}^{0}$ and (**d**) current density $J_{1}\;A/m^{2}$ for the maximum $\Pi_{11}^{Eff}$ case at 0.24% CNT volume fraction for the randomly oriented case.

The deviation found in the data sets of the piezoresistive terms can be studied with the help of a histogram together with the corresponding maximum effective case, which was done in the initial study [8] for the aligned case. In the present study, the randomly oriented case is investigated. Figure 23a shows the histogram distribution for $\Pi_{11}^{Eff}$ values obtained at 0.24% CNT volume fraction for the randomly oriented case. The coefficient of variation for the $\Pi_{11}$ data set at this volume fraction is the highest among all the volume fractions studied, making it a good selection for illustration purposes. The selected distribution is right skewed with a skewness value of 2.04 and kurtosis value of 2.38. These values are similar to the values observed for $\Sigma_{11}$ and $\Sigma_{22}$ in Figure 17 and Figure 18 within the PTR. The right skew of the distribution suggests that most of the data corresponds to unpercolated realizations with a few of the data points corresponding to percolates ones. However, some of the realizations do exhibit percolation which give rise to high values of $\Pi_{11}^{Eff}$. For the realization corresponding to the highest $\Pi_{11}^{Eff}$ at the volume fraction of 0.24%, Figure 23 shows local electrical conductivity $\Sigma_{11}$, local relative change in resistivity $\Delta \rho /\rho_{1}^{0}$ and local current density $J_{1}$. From Figure 23b, it can be observed that the dispersion of CNTs occur in a favorable way such that a network path is created across the realization. This leads to percolation in the ${\tilde{x}}_{1}$ direction. Figure 23d, which shows the current density contours, confirms this since current is seen to be primarily flowing through this conductive path. With mechanical loading being applied (i.e., test 1), the CNTs and their corresponding electrical tunneling clouds separate out from each another (as seen in Figure 23c). This results in the intermediate junction areas between these tunneling clouds exhibiting relatively large values of local relative change in resistivity $\Delta \rho /\rho_{1}^{0}$. Thus, the loading results in a substantial change in the current carrying capacity of this network path, thus producing a high $\Pi_{11}^{Eff}$ value of 16.40. Since all other $\Pi_{11}^{Eff}$ values are significantly lower than 16.40, it is clear that most realizations result in unfavorable CNT dispersions that fail to lead to percolation in the ${\tilde{x}}_{1}$ direction at 0.24% CNT volume fraction.

**Figure 24:**
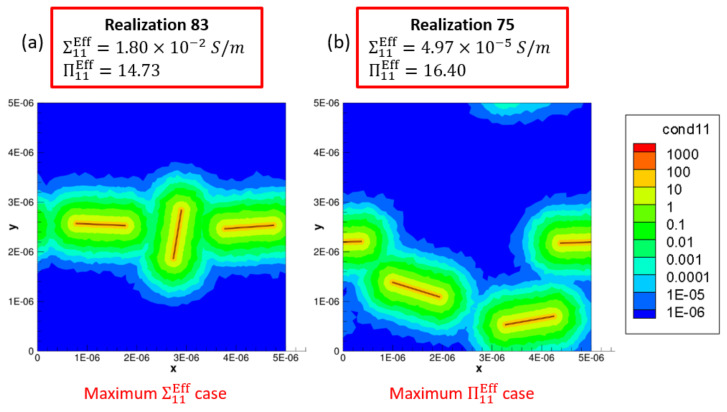
Local conductivity $\Sigma_{11}\;S/m$ for the (**a**) maximum $\Sigma_{11}^{Eff}$ and (**b**) maximum $\Pi_{11}^{Eff}$ cases at 0.24% CNT volume fraction for randomly oriented orientation condition.

It is worth noting that the CNT dispersion which produced the highest $\Pi_{11}^{Eff}$ is not the same dispersion that produces the highest $\Sigma_{11}\;S/m$. This can be observed in Figure 24 which shows the conductivity contours for both of these cases. Figure 24a is repeated from Figure 23b for comparison sake. The highest conductivity case belongs to a group of cases demonstrating network redundancy, one of the significant factors that dictate the piezoresistivity. Figure 24a shows that the CNT dispersion has been randomly selected in such a way that there is a strong conductive network across the realization in the ${\tilde{x}}_{1}$ direction with tunneling clouds of the CNTs overlapping each other to a relatively larger extent than the maximum $\Pi_{11}^{Eff}$ case observed in Figure 24b. Thus, when mechanical loading is applied (test 1), the electrical tunneling clouds for the maximum $\Pi_{11}^{Eff}$ case will separate and therefore overlap less. The disruption caused to the current flow is to larger extent than the maximum $\Sigma_{11}^{Eff}$ case where at the same deformation the dispersion retains a very large degree of conductive region overlap. Finally, as volume fraction increases, the number of realizations that exhibit this type of network redundancy increases and this is consistent with the observed electrical percolation transition behavior.

**Figure 25:**
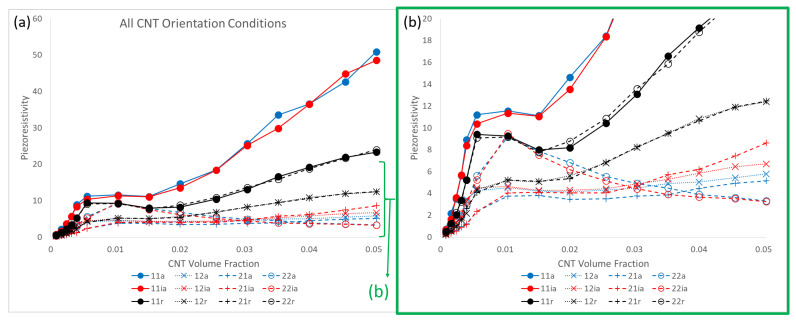
(**a**) Estimate of all non-zero piezoresistivity terms for all orientation conditions with (**b**) a zoomed in version.

In the initial study [8], it was found that the estimates for piezoresistive components $\Pi_{16}$, $\Pi_{26}$, $\Pi_{61}$, $\Pi_{62}$ and $\Pi_{66}$ were all insignificantly small values for all simulated CNT volume fractions for the aligned case. Similarly, these piezoresistive components are found to be near zero values even for the ${\tilde{x}}_{1}$ imperfectly aligned and randomly oriented cases and are therefore assumed as zero at all CNT volume fractions. The estimates for $\Pi_{11}$, $\Pi_{12}$, $\Pi_{21}$ and $\Pi_{22}$ are presented in Figure 25 for all orientation conditions. Between the ${\tilde{x}}_{1}$ aligned and ${\tilde{x}}_{1}$ imperfectly aligned cases, each piezoresistive component is approximately the same for all simulated CNT volume fractions, with the exception of $\Pi_{12}$ and $\Pi_{21}$ being slightly larger for the ${\tilde{x}}_{1}$ imperfectly aligned case than for the ${\tilde{x}}_{1}$ aligned case at higher CNT volume fractions. This exception is a result of the slight misalignment making it more conducive for the inherent CNT piezoresistivity effect to manifest into larger piezoresistivity estimates for the $\Pi_{12}$ and $\Pi_{21}$ components. $\Pi_{11}$ estimate reaches relatively higher values for the alignment cases. This is expected since the direction of CNT alignment aids in the piezoresistive effect in that direction. $\Pi_{22}$ is larger than the off-diagonal terms $\Pi_{12}$ and $\Pi_{21}$ up until a volume fraction of around 3.52%. Beyond this volume fraction, the trend reverses due to effect of network redundancy (affecting $\Pi_{22}$ strongly) and the effect of inherent CNT piezoresistivity boosting the $\Pi_{12}$ and $\Pi_{21}$ estimates.

The curves for $\Pi_{11}$ and $\Pi_{22}$ in Figure 25 for the randomly oriented case are almost identical to each other, with the curves lying between the $\Pi_{11}$ and $\Pi_{22}$ curves for the ${\tilde{x}}_{1}$ aligned and ${\tilde{x}}_{1}$ imperfectly aligned cases. The initial peak (at the boundary between region B and region C) for $\Pi_{11}$ and $\Pi_{22}$ for the randomly oriented case is at a lower value of 9.41 compared to $\Pi_{11}$ for the two alignment cases. $\Pi_{12}$ and $\Pi_{21}$ for the randomly oriented case are also almost identical to each other. In addition, $\Pi_{12}$ and $\Pi_{21}$ for the randomly oriented case are consistently larger than those for the two alignment cases in region D due to the random orientation of CNTs being more conducive for the inherent CNT piezoresistivity effect to influence the effective properties, i.e., a combination of both factors being partially satisfied appears to have a more pronounced effect than fully satisfying either factor. The following points may be enumerated as the key factors controlling the piezoresistive effect for CNT/polymer nanocomposites:

- CNT volume fraction.
- CNT dispersion and probability of percolation.
- Probability of network redundancy.
- Mechanical deformation resulting in changes in the current carrying capacity, which varies based on loading direction (${\tilde{x}}_{1}$ vs. ${\tilde{x}}_{2}$ directions depending upon the orientation condition).
- CNT inherent piezoresistivity, which depends upon mechanical loading and thereby the loading direction (${\tilde{x}}_{1}$ vs. ${\tilde{x}}_{2}$ directions depending upon the orientation condition).
- Orientation condition.

#### 3.3.2. Comparison to Other Studies in the Literature

Equations 11 and 12 provide the macroscale gage factors for CNT/polymer nanocomposites in the ${\tilde{x}}_{1}$ and ${\tilde{x}}_{2}$ directions, respectively. The piezoresistivity estimates are used in these equations. All simulations conducted in this work utilize a low applied strain of $10^{-4}$ (${\tilde{\varepsilon }}_{0}<<1$). Therefore, the gage factors $G_{1}$ and $G_{2}$ reduce to $\Pi_{11}+1$ and $\Pi_{22}+1$, respectively. The initial study [8] focused on comparing estimates obtained for the aligned case with other characterization efforts in the literature. Here, the estimates from the more generic randomly oriented case will be used for comparison with other case studies. The increase in the gage factors can be visualized with Figure 25b, where $G_{1}$ and $G_{2}$ can be approximated as $\Pi_{11}+1$ and $\Pi_{22}+1$, respectively.

**Figure 26:**
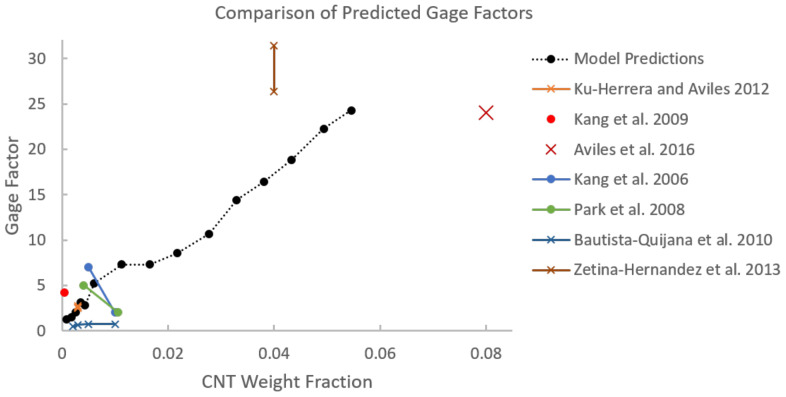
Comparing gage factors for randomly oriented straight CNTs from modeling predictions with characterization efforts in the literature [9,59,60,61,62,63,64].

From the present study, gage factors $G_{1}$ and $G_{2}$ increase non-linearly from unity at 0% CNT volume fraction up to a value of approximately 10 at a CNT volume fraction of 0.56% (∼ 0.61 wt.%) for randomly oriented CNTs. The comparisons of the gage factor modeling predictions for randomly oriented straight CNTs with other studies found in the literature is summarized in Figure 26, where a good match is observed. Several works are referenced in Figure 26, including Ku-Herrera and Aviles [59], who measured tensile gage factors for dogbone specimens for 0.3 wt.% multi-walled CNT/vinyl ester nanocomposite and obtained a value of 2.6 ± 0.13. Similarly, Kang et al. [60] measured the piezoresistive effect of single-walled CNT/polyimide nanocomposite and obtained a maximum gage factor magnitude of 4.21 at ∼ 0.05 CNT wt.%.

For CNT volume fractions beyond percolation, the model predicts gage factors as high as 20 at 5.04% CNT volume fraction. This is also observed in some works referenced in Figure 26. For example, Zetina-Hernàndez et al. [64] showed a gage factor of 28.9 ± 2.55 at 4 wt.% CNT for CNT/polypropylene composites. Similarly, Aviles et al. [61] obtained a gage factor as high as 24 for multi-walled CNTs with a high aspect ratio and CNT weight concentration of 8% in a polypropylene matrix.

The model predictions point to a decrease in the gage factor values after an initial peak due to network redundancy, i.e., region C. Effective gage factor drops from 10.4 to 8.98 from 0.56% to 1.52% CNT volume fraction, respectively. This drop is also found in some studies referenced in Figure 26. For example, Kang et al. [9] used an electrical model of a nanotube strain sensor to study single-walled CNT buckypaper and single-walled CNT PMMA sensors, and reported a decrease in gage factor from 5 to 1 with an increase in the CNT wt.% from around 0.5% to 1.0%. Similarly, Park et al. [62] reported gage factor values of 5 and 2, respectively, for 0.56 vol.% and 1.44 vol.% multi-walled CNT reinforcement embedded in polyethylene oxide.

### 3.4. Electrical and Mechanical Properties with Wavy CNTs

#### 3.4.1. Engineering Constants

**Figure 27:**
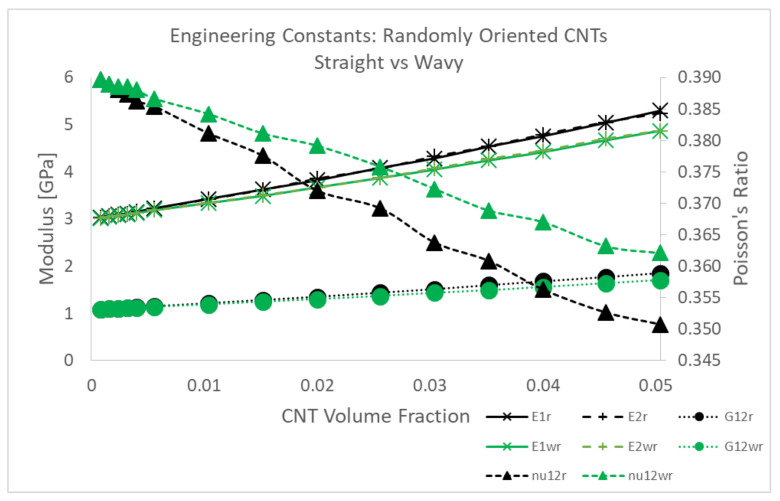
Comparison of engineering constant estimates $E_{1}$, $E_{2}$, $G_{12}$ and $\nu_{12}$ between the randomly oriented straight case (r) and randomly oriented wavy case (wr).

The estimate plots showing the effective $C_{11}$, $C_{22}$ and $C_{66}$ for the randomly oriented wavy case are shown in the Supplementary Materials. These mechanical stiffness estimates for the randomly oriented wavy case can be converted to engineering constants in the same way as was done for the randomly oriented straight case using Equation 14. The estimates for the engineering constants $E_{1}$, $E_{2}$, $\nu_{12}$ and $G_{12}$ are presented along with the corresponding estimates for the randomly oriented straight case in Figure 27, from which it can clearly be observed that the tensile moduli $E_{1}$ and $E_{2}$ and shear modulus $G_{12}$ are consistently smaller for the randomly oriented wavy case compared to the randomly oriented straight case. Furthermore, the properties in the ${\tilde{x}}_{1}$ or ${\tilde{x}}_{2}$ direction are very similar with $E_{1}$ and $E_{2}$ being almost identical to each other other at every CNT volume fraction since the CNTs are randomly oriented in this case. At the final simulated CNT volume fraction of 5.04%, $E_{1}$, $E_{2}$ and $G_{12}$ are 8.72%, 7.60% and 8.75% smaller for the wavy case compared to the straight case (4.871 GPa vs. 5.297 GPa for $E_{1}$, 4.869 GPa vs. 5.239 GPa for $E_{2}$ and 1.707 GPa vs. 1.856 GPa for $G_{12}$ in terms of wavy vs. randomly oriented straight). This behavior is consistent with some other modeling works found in the literature which also predict a decrease in the stiffness properties of CNT/polymer nanocomposites with the introduction of CNT waviness. For example, Herasati and Zhang [65] found that the increase in the waviness angle caused a decrease in the elastic properties using a finite element model based on a segmented CNT generation process to account for waviness. Dastgerdi et al. [21], using a model based on micromechanics, showed that with the increase in the degree of CNT waviness, the effective elastic modulus of CNT/shape memory polymer composites can reduce from 9 GPa to as low as 1 GPa at a CNT volume fraction of 0.1. For the estimate of Poisson’s ratio $\nu_{12}$, it is observed that the wavy case has a lower rate of decrease with increasing CNT volume fraction with $\nu_{12}$ at the final simulated volume fraction of 5.04% being 0.362 for the wavy case as opposed to 0.351 for the randomly oriented straight case (increase of 3.04% with the inclusion of waviness).

Overall, it can be concluded that the effect of CNT waviness on the mechanical properties is marginal compared to the effect of alignment. This can be verified by observing the evolution of $E_{1}$ with respect to CNT volume fraction in Figure 10 and Figure 27 for straight ${\tilde{x}}_{1}$ aligned vs. straight random comparison and straight random vs. wavy random comparison, respectively. For the first comparison, the largest difference in effective $E_{1}$ is 68.29% at 5.04% CNT volume fraction. On the other hand, the second comparison between wavy and straight CNTs with random orientation reveals a largest difference value in effective $E_{1}$ of only 15.16%.

#### 3.4.2. Comparing Initial Electrical Conductivity

**Figure 28:**
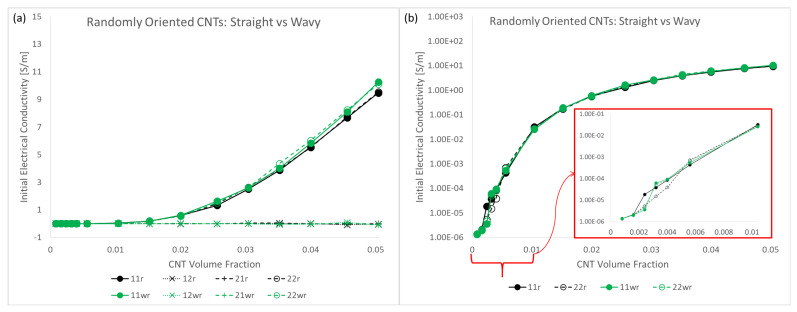
(**a**) Mean of all initial electrical conductivity terms $\Sigma_{ij}$ and (**b**) mean of $\Sigma_{11}$ and $\Sigma_{22}$ on a semi-log scale for the randomly oriented straight (r) and randomly oriented wavy cases (wr).

The estimate plots and the semi-log plots for $\Sigma_{11}$ and $\Sigma_{22}$ for the randomly oriented wavy case are shown in the Supplementary Materials. Figure 28 presents the conductivity components for the randomly oriented wavy and randomly oriented straight cases. From the inset of the semi-log plot in Figure 28b, it is observed that in the beginning portion of the PTR (between 0.24% and 0.56% CNT volume fractions), the trend fluctuates and this is because of the highly skewed nature of the distributions found at these volume fraction with high skewness and kurtosis values. It is observed that the evolution of $\Sigma_{11}$ and $\Sigma_{22}$ in the wavy case are very similar to the straight case. Furthermore, $\Sigma_{11}$ and $\Sigma_{22}$ are approximately equal to one another for all CNT volume fractions for the wavy case, which should be expected since there is no bias to the CNT seeding process in any direction for randomized orientation. At the final CNT volume fraction of 5.04%, $\Sigma_{11}$ and $\Sigma_{22}$ for the wavy random case are 8.36% and 6.07% larger than their respective straight random counterpart. In contrast, at the same volume fraction, $\Sigma_{11}$ and $\Sigma_{22}$ for the ${\tilde{x}}_{1}$ aligned case are 32.54% larger and 85.38% smaller than their respective straight random counterpart. Thus, the effect of waviness is marginal compared to the effect of alignment and this can be visualized with Figure 20. From Figure 28 it can be confirmed that the conductivity components $\Sigma_{12}$ and $\Sigma_{21}$ are near zero values for both cases.

It is speculated that post the PTR, where percolation of any given realization is almost guaranteed, the efficiency of the conductive channels improves stochastically as a result of CNT waviness due to increased probability and efficiency (minimum distance between CNTs being smaller) of conductive junctions between the highly conductive CNT phases, i.e., the orientation distribution of CNT segments overall increased for the wavy cases compared to the straight cases. This means that with straight CNTs there are only *N* number of states that a CNT can exist in in terms of its morphology dictated only by its orientation, but with wavy CNTs there are many more number of states that a CNT can exist in on the order of $N^{2}$ as a result of its orientation as well as the orientation of individual segments of the CNT which is informed by the waviness function.

#### 3.4.3. Comparing Nanocomposite Piezoresistivity

**Figure 29:**
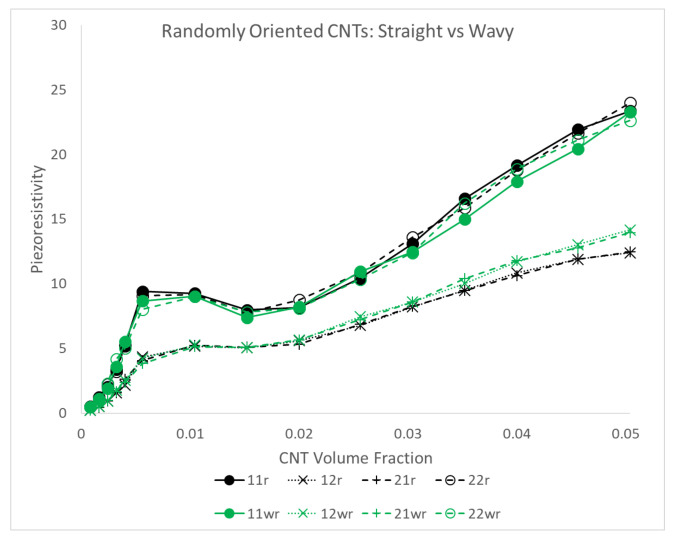
Mean of all non-zero piezoresistivity terms for the randomly oriented straight and randomly oriented wavy cases.

The estimate plots showing the effective non-zero components of the piezoresistivity tensor for the randomly oriented wavy case are shown in the Supplementary Materials. Comparisons to the randomly oriented straight cases are presented in Figure 29.The estimates for the non-zero components of the piezoresistivity tensor for the wavy and randomly oriented straight cases are presented in Figure 29. (The piezoresistive coefficients $\Pi_{16}$, $\Pi_{26}$, $\Pi_{61}$, $\Pi_{62}$ and $\Pi_{66}$ all exhibit scatter in their respective data sets with mean values close to zero at all CNT volume fractions for the wavy case just like all the other cases in this study with straight CNTs). There is no well defined ordering for the piezoresistive components $\Pi_{11}$ and $\Pi_{22}$ when making comparisons between the wavy and the randomly oriented straight cases. At the higher end of the simulated CNT volume fraction range beyond the PTR (≥2.00%), $\Pi_{12}$ and $\Pi_{21}$ are larger for the wavy case than the randomly oriented straight case. Similar to the higher conductivity estimates obtained for the wavy case, this may be explained with the extra pathways obtained in the wavy cases due to the increased orientation distribution of CNT segments. Overall, it is observed that the effect of waviness is marginal compared to the effect of alignment (in terms of ${\tilde{x}}_{1}$ aligned vs. randomly oriented in Figure 25) similar to the estimates obtained for engineering constants and conductivity components for the wavy case.

The similarity in the mechanical, electrical and piezoresistive properties between the randomly oriented wavy case and randomly oriented straight case means that the comparisons made for the randomly oriented straight case with experimental characterization efforts found in the literature can be extended to the wavy case as well. In fact, the randomly oriented wavy case is the more generic case that more accurately captures the actual morphology of CNTs within well dispersed CNT/polymer nanocomposite samples. As such, the modeling predictions made for the most representative case (randomly oriented CNTs with waviness) match well with the electro-mechanical properties found in the literature for CNT/polymer nanocomposites, including the predictions made with respect to the phenomenon of electrical percolation.

## 4. Summary of Results

A summary of the most significant results from the present study are as follows (recalling that the alignment direction for the aligned and imperfectly aligned cases is the ${\tilde{x}}_{1}$ direction):

- Mechanical reinforcement in the ${\tilde{x}}_{1}$ direction increased with the degree of alignment (${\tilde{x}}_{1}$ aligned >${\tilde{x}}_{1}$ imperfectly aligned > randomly oriented) for all CNT volume fractions. On the other hand, mechanical reinforcement in the ${\tilde{x}}_{2}$ direction increased with the degree of misalignment (randomly oriented >${\tilde{x}}_{1}$ imperfectly aligned >${\tilde{x}}_{1}$ aligned) for all CNT volume fraction. This was found to be true for both the stiffness terms $C_{11}$ and $C_{22}$ and for the engineering constants $E_{1}$ and $E_{2}$. With increasing CNT volume fraction, the difference in the stiffness estimates of the aligned case with respect to the randomly oriented case grew larger. At the final simulated CNT volume fraction of 5.04%, this difference for $E_{1}$ was 72.80% and for $E_{2}$ was −15.16%.
- The estimates for the initial electrical conductivity terms $\Sigma_{11}$ and $\Sigma_{22}$ increased by several orders of magnitude to values higher than $10^{1}\;S/m$ with increasing CNT volume fraction. The enhancement in $\Sigma_{11}$ was found to be consistently larger with increase in the degree of alignment and vice versa for $\Sigma_{22}$ for all CNT volume fractions. At the final simulated CNT volume fraction of 5.04%, $\Sigma_{11}$ and $\Sigma_{22}$ for the aligned case were 32.54% larger and 85.38% smaller than their corresponding values for the randomly oriented case.
- Percolation transition regions or PTR were determined using a threshold measure. PTR varied from one orientation condition to the next and was also different in the ${\tilde{x}}_{1}$ and ${\tilde{x}}_{2}$ directions for the ${\tilde{x}}_{1}$ aligned and ${\tilde{x}}_{1}$ imperfectly aligned cases. The transitional behavior of the electrical percolation phenomena was co-related with histograms and skewness-kurtosis plots.
- The piezoresistive terms evolved in a more complicated manner than the stiffness or conductivity terms. Piezoresistivity was found to depend upon a number of different factors such as (i) CNT volume fraction, (ii) CNT dispersion, (iii) network redundancy, and (iv) inherent CNT piezoresistivity. The effect of piezoresistivity was found to be stronger in the ${\tilde{x}}_{1}$ direction ($\Pi_{11}$) with increasing alignment (${\tilde{x}}_{1}$ aligned >${\tilde{x}}_{1}$ imperfectly aligned > randomly oriented) and vice versa for the ${\tilde{x}}_{2}$ direction ($\Pi_{22}$). The largest difference in values for $\Pi_{11}$ and $\Pi_{22}$ for aligned case with respect to the randomly oriented case is found at 5.04% CNT volume fraction, with values of 117.91% and −86.21%, respectively.
- The effect of CNT waviness proved to be insignificant when compared to the effects of alignment evidenced by the similarity in the obtained electro-mechanical properties for the randomly oriented straight case and the randomly oriented wavy case.
- Comparing the percolation transition region for the randomly oriented straight CNTs using three different barrier potential values of $\lambda =10^{-6}\;\mathrm{eV}$, $\lambda =10^{-5}\;\mathrm{eV}$ and $\lambda =10^{-4}\;\mathrm{eV}$ with experimentally reported values of percolation threshold revealed that the range from $\lambda =10^{-6}\;\mathrm{eV}$ case matched the observations more closely.
- All electro-mechanical properties obtained for the randomly oriented straight and randomly oriented wavy cases exhibited isotropy, i.e., confirming the unbiased nature of the algorithm employed for generating nanocomposite realizations.

## 5. Conclusions

The electrical and mechanical properties of CNT/polymer nanocomposites have been studied with different orientation conditions. Effective estimates and the statistical distribution in the data were studied by generating a library of realizations at several CNT volume fractions. A finite element based numerical code was employed together with a tunneling resistivity model to simulate the phenomenon of electrical tunneling. CNT volume fractions up till 5.04% were studied. A parallelized computational framework was developed to analyze the vast number of realizations generated. The statistical analysis approach employed in this study is of merit since effective properties are highly sensitive to inhomogeneity dispersions in composites.

Modeling predictions revealed that alignment played a strong role in the enhancement of the electrical and mechanical properties. With increase in CNT volume fraction, percolation transition regions were observed, which were regions with orders of magnitude increase in the effective electrical conductivity. These regions exhibited a high degree of variance in their effective electrical properties because of the similar probabilities of obtaining a percolated or unpercolated realization due to randomized seeding. CNT waviness was incorporated into the developed model by mapping randomly generated waviness functions. The role of waviness was found to be marginal in the predicted properties. Contrasting the results from the randomly oriented case with several studies found in the literature revealed good agreement, thereby validating the developed model herein.

## Figures and Tables

**Table 1:** CNT and polymer isotropic homogeneous material properties (E: Young’s modulus, ν: Poisson’s ratio, $\Sigma_{ij}$: components of electrical conductivity, Π: nanoscale effective piezoresistivity coefficient).

Pure matrix (no electron hopping): Epoxy	
E = 3.00 GPa	ν = 0.39
$\Sigma_{ij}=10^{-6}\;S/m$ if i=j	$\Sigma_{ij}=0\;S/m$ if i≠j
Π=0	
**Multi-walled carbon nanotube**	
E = 900 GPa	ν = 0.3
CNT Length = 1 μm	CNT Diameter = 20 nm
$\Sigma_{ij}=10^{3}\;S/m$ if i=j	$\Sigma_{ij}=0\;S/m$ if i≠j
$\Pi =1.289\times 10^{7}$	
**Barrier Potential**	
$\lambda =10^{-6}\;\mathrm{eV}$	

## Data Availability

The data presented in this study are available on request from the corresponding author. The data are not publicly available due to ongoing future work.

## Supplementary Materials

The following supporting information can be downloaded at: https://drive.google.com/file/d/1RaInqJAjD9QSOnORuHLPHe4zTn5tUfcP/view?usp=share_link.
